# Tandem metalloenzymes gate plant cell entry by pathogenic fungi

**DOI:** 10.1126/sciadv.ade9982

**Published:** 2022-12-21

**Authors:** Bastien Bissaro, Sayo Kodama, Takumi Nishiuchi, Anna Maria Díaz-Rovira, Hayat Hage, David Ribeaucourt, Mireille Haon, Sacha Grisel, A. Jalila Simaan, Fred Beisson, Stephanie M. Forget, Harry Brumer, Marie-Noëlle Rosso, Victor Guallar, Richard O’Connell, Mickaël Lafond, Yasuyuki Kubo, Jean-Guy Berrin

**Affiliations:** ^1^ INRAE, Aix Marseille Université, UMR1163 Biodiversité et Biotechnologie Fongiques, 13009 Marseille, France.; ^2^ Faculty of Agriculture, Setsunan University, 573-0101 Osaka, Japan.; ^3^ Division of Functional Genomics, Advanced Science Research Center, Kanazawa University, 920-0934 Kanazawa, Japan.; ^4^ Barcelona Supercomputing Center, Plaça Eusebi Güell, 1-3, E-08034 Barcelona, Spain.; ^5^ Aix Marseille Université, CNRS, Centrale Marseille, iSm2, Marseille, France.; ^6^ V. Mane Fils, 620 route de Grasse, 06620 Le Bar sur Loup, France.; ^7^ CEA, CNRS, Aix Marseille Université, Institut de Biosciences et Biotechnologies d’Aix-Marseille (UMR7265), CEA Cadarache, 13108 Saint-Paul-lez-Durance, France.; ^8^ Michael Smith Laboratories, University of British Columbia, 2185 East Mall, Vancouver, BC V6T 1Z4, Canada.; ^9^ ICREA, Passeig Lluís Companys 23, E-08010 Barcelona, Spain.; ^10^ INRAE, UMR BIOGER, AgroParisTech, Université Paris-Saclay, Thiverval-Grignon, France.

## Abstract

Global food security is endangered by fungal phytopathogens causing devastating crop production losses. Many of these pathogens use specialized appressoria cells to puncture plant cuticles. Here, we unveil a pair of alcohol oxidase–peroxidase enzymes to be essential for pathogenicity. Using *Colletotrichum orbiculare*, we show that the enzyme pair is cosecreted by the fungus early during plant penetration and that single and double mutants have impaired penetration ability. Molecular modeling, biochemical, and biophysical approaches revealed a fine-tuned interplay between these metalloenzymes, which oxidize plant cuticular long-chain alcohols into aldehydes. We show that the enzyme pair is involved in transcriptional regulation of genes necessary for host penetration. The identification of these infection-specific metalloenzymes opens new avenues on the role of wax-derived compounds and the design of oxidase-specific inhibitors for crop protection.

## Teaser

Fungal phytopathogens secrete tandem metalloenzymes that catalyze cuticle oxidation and drive plant cell entry.

## INTRODUCTION

**Fig. 1.**
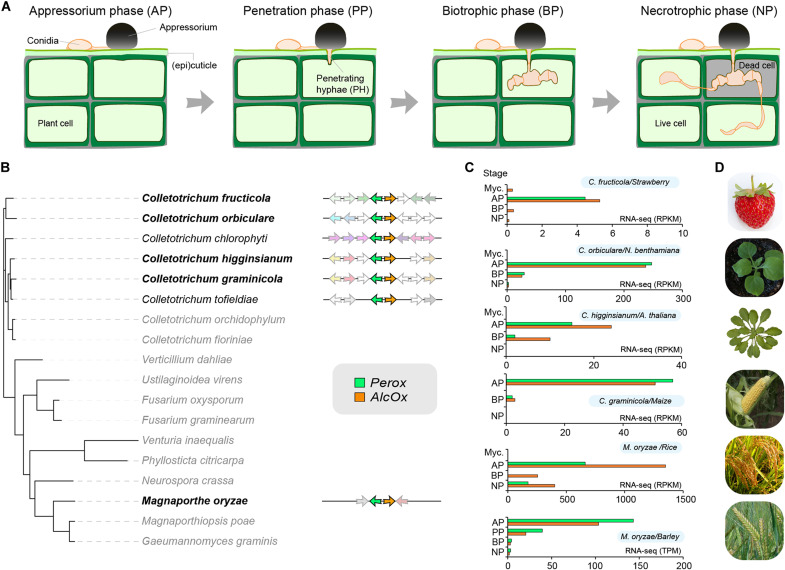
Genomic and transcriptomic analysis of the Perox-AlcOx pair. (**A**) Multistage plant infection process of appressorium-forming fungi: appressorium (AP), penetration (PP), biotrophic (BP), and necrotrophic (NP) phases. (**B**) Phylogenomic occurrence of the *perox-alcox* pair and consensus genomic environment (within each clade) among pathogenic ascomycetes. Black bold lettering indicates species for which transcriptomics data are shown in (C), and gray lettering indicates the absence of the gene pair. Note that analogous tandem oxidase systems may occur in other fungi but were possibly not detected because of the stringent sequence identity thresholds used to define AlcOx- and Perox-encoding genes. Selected *Colletotrichum* species and associated consensus sequences are representative of their respective species complexes (see fig. S3). (**C**) Time-course transcriptomic analysis of the Tandem Peroxidases (Perox, green)– and AlcOx (orange)–encoding genes (gene accession numbers in table S1) during plant infection for different pathosystems (*26*–*29*). Myc., mycelium. Actual time points associated with each infection stages are provided in Materials and Methods. (**D**) Illustration of targeted plant hosts.

Fungal phytopathogens represent a serious threat to plant health (*1*) and global food security (*2*). *Colletotrichum* and *Magnaporthe* species rank among the top 10 most devastating fungal phytopathogens in the world and reduce crop yield by up to 30% (*3*). Despite being separated by ca. 300 million years of evolution (*4*, *5*), these fungi share remarkable similarities in their infection strategy, notably the formation of a specialized cell dedicated to host penetration called an appressorium (Fig. 1A) (*6*–*8*). This dome-shaped, darkly melanized cell generates a high internal turgor and directs this mechanical pressure onto a needle-like penetration peg (*9*–*11*), which emerges from a 2- to 500-nm pore at the appressorial base to puncture the plant’s outer defensive barriers, namely, the cuticle and epidermal cell wall. Despite major advances in our understanding of the cellular processes preceding (*6*, *7*, *11*–*14*) and following (*15*, *16*) plant cell entry, the (bio)chemical reactions occurring at this nanoscale plant-fungus interface and their role in host penetration are not fully elucidated.

Copper radical oxidases (CROs) are enzymes with diverse substrate specificities and have been extensively studied since the late 1960s (*17*). Today, CROs include galactose 6-oxidases (GalOx) (*17*), glyoxal oxidases (*18*), and broad specificity primary alcohol oxidases (AlcOx) (*19*, *20*). Despite the detailed knowledge available on the enzymology and structure of CROs, their biological function remains largely unknown. It has only been proposed that glyoxal oxidases play a role in lignin degradation by fungal saprotrophs (*18*). During the course of our previous work (*19*), we noted that genes encoding secreted AlcOx orthologs are particularly widespread among phytopathogenic ascomycete fungi and are absent in plants. Given that long-chain primary alcohols are components of the waxy cuticle of aerial plant surfaces (*21*), we hypothesized that AlcOx could play a role in fungal pathogenesis.

In this study, we used a combination of ‘omics analyses to unveil the pairing of AlcOx with a redox partner, namely, heme-peroxidase, in some fungal plant pathogens of high agricultural importance. We used wet enzymology and molecular modeling to demonstrate and characterize the interplay between these metalloenzymes. Reverse genetics, live-cell imaging, and fungal transcriptomics allowed us to probe the in vivo function of the enzyme pair, providing new molecular insights into the host penetration cascade.

## RESULTS

### Discovery of the tandem Perox-AlcOx

While studying the enzymology of *Colletotrichum* AlcOx enzymes for biotechnological applications (*19*, *22*), we noticed the presence of a gene encoding a putative peroxidase located adjacent to an AlcOx-encoding gene (Fig. 1B). To strengthen this initial observation, we searched for *alcox* orthologs in 30 sequenced *Colletotrichum* genomes, which revealed the near-ubiquitous presence of a putative peroxidase (hereafter called “Tandem Peroxidase”). The *perox* and *alcox* genes were found in a head-to-head arrangement (fig. S1), suggesting the presence of a bidirectional promoter for tight coexpression of the genes. These Tandem Peroxidases are never found in combination with other types of CROs (fig. S1C), and both proteins encoded by the *perox-alcox* pair are predicted to be secreted (table S1). These observations aroused our interest because it is known that CROs require activation by horseradish peroxidase (HRP) for maximum activity in vitro (*23*, *24*).

Our phylogenetic analysis of the peroxidase-catalase superfamily showed that the Tandem Peroxidases cluster together in a sister clade within the under-explored ascomycete class II peroxidases (fig. S2A) (*25*). Furthermore, Tandem Peroxidases form a distinct clade among the 333 class II peroxidases found in *Colletotrichum* species (fig. S2B), suggestive of neofunctionalization. A broad search for the cooccurrence of *perox* and *alcox* orthologs across fungal genomes revealed that the pair is also present in *Magnaporthe* species, including the infamous causal agent of rice blast, *Magnaporthe oryzae* (syn. *Pyricularia oryzae*) (table S2). Mapping the occurrence of Perox-AlcOx protein pairs and their corresponding genomic neighborhoods onto a phylogeny of representative pathogenic ascomycetes (Fig. 1B and fig. S3) allowed us to conclude that the pair is present in most *Colletotrichum* species complexes for which genome sequences are available and in *Magnaporthe spp.*, and that the head-to-head organization of the pair is conserved in all these fungi, suggesting that there is selection pressure to retain the pairing and that it has a critical role in the biology of these pathogens (Fig. 1B).

To further test the hypothesis of a functional linkage of the *perox* and *alcox* gene products, we parsed transcriptomic data available for those fungal species harboring the pair. This analysis revealed that both genes are always tightly cotranscribed at the appressorium stage in various pathosystems involving *Colletotrichum* species attacking maize, fruits, and model plants (*26*, *27*) and in *M. oryzae* attacking rice and barley (Fig. 1, C and D) (*28*, *29*). In each case, the transcript levels are relatively low and detected within a narrow time window, which may explain why these genes were overlooked in previous studies.

### Tandem Perox-AlcOx oxidize plant long-chain alcohols

**Fig. 2.**
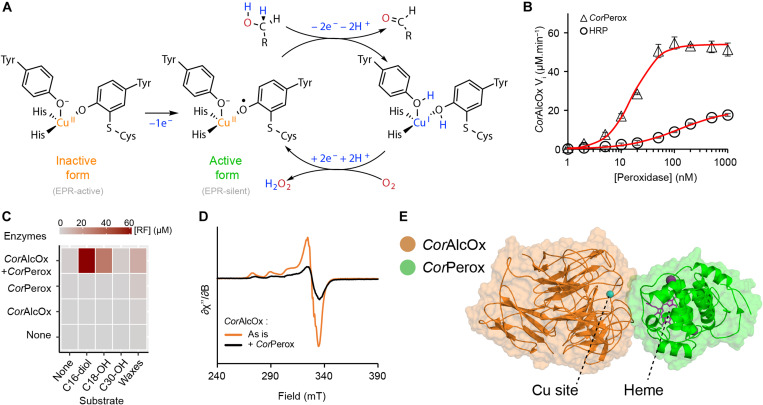
Biochemical and biophysical evidence for interplay between *Cor*Perox and *Cor*AlcOx. (**A**) Reaction mechanism of CROs showing activation of the resting, inactive form of the enzyme via formation of a tyrosine radical, yielding the Cu(II)-radical active form. The latter will oxidize an alcohol into the corresponding aldehyde followed by regeneration of the active form via the two-electron reduction of O_2_ into H_2_O_2_. (**B**) *Cor*AlcOx oxidation rate of benzyl alcohol in the presence of varying amounts of HRP or *Cor*Perox. (**C**) Activity of *Cor*Perox-*Cor*AlcOx on crude extract of cucumber waxes and derived long-chain aliphatic alcohols, monitored via the production of chromogenic resorufin (RF), product of the Perox-catalyzed oxidation of Amplex Red by H_2_O_2_, the latter being the coproduct of AlcOx-catalyzed oxidation of primary alcohols into aldehydes. (**D**) EPR spectra of inactive *Cor*AlcOx before (orange curve) and after mixture with *Cor*Perox (black curve). EPR parameters of the Cu(II) inactive form: *g*_z_ = 2.270, $A_{z}^{\mathrm{Cu}}$ = 171 × 10^−4^ cm^−1^, *g*_x_ = 2.047, $A_{x}^{\mathrm{Cu}}$ < 50 × 10^−4^ cm^−1^, *g*_y_ = 2.054, $A_{y}^{\mathrm{Cu}}$ < 50 × 10^−4^ cm^−1^, and super-hyperfine coupling constant corresponding to two N-ligands *A*^N^ = 43 × 10^−4^ cm^−1^. (**E**) Lowest-energy *Cor*AlcOx-*Cor*Perox complex obtained by protein-protein modeling simulation with PELE (see fig. S6 for more details). The copper atom is shown as a blue sphere, the heme group is shown as magenta sticks, and calcium ions are shown as purple spheres.

As a prelude to analyzing their biological function in vivo, we studied the substrate specificity and enzyme interplay of the Perox-AlcOx pair in vitro (Fig. 2A). *Colletotrichum orbiculare* was selected as a model because it not only causes the economically important anthracnose disease of cucurbits (e.g., melons and cucumber) but also has been used for decades as a model system for studying fungal pathogenesis (*30*). Despite the notorious difficulties associated with heterologous expression of these metalloenzymes, we successfully produced in the yeast *Pichia pastoris* recombinant copper radical AlcOx and heme iron tandem peroxidase from *C. orbiculare* (hereafter *Cor*AlcOx and *Cor*Perox, respectively). Similar to the previously studied AlcOx orthologs from *Colletotrichum graminicola* and *Colletotrichum gloeosporioides* (*19*) (fig. S1A), *Cor*AlcOx oxidized both aromatic and long-chain aliphatic primary alcohols (fig. S4A). This finding raises the possibility that fatty primary alcohols present in the cuticle of many plant species (*21*), including cucumber, could be the native substrates of these enzymes.

On the other hand, *Cor*Perox was confirmed to be a peroxidase, albeit with moderate catalytic efficiency (*k*_cat_ = 1.52 ± 0.02 s^−1^, *K*_M_^H2O2^ = 80 ± 3 μM, *k*_cat_/*K*_M_ = 1.9 × 10^4^ ± 0.1 s^−1^ M^−1^) compared to the only previously characterized ascomycete class II peroxidase (*25*), commercial HRP (*31*), and well-studied basidiomycete lignin-active peroxidases (*32*) (*k*_cat_/*K*_M_ = 10^4^ to 10^7^ s^−1^ M^−1^). *Cor*Perox was only active on low redox potential substrates and not on any of the substrates of canonical lignin-active peroxidases (fig. S4B) and required the presence of calcium ions for stability (fig. S4C). These observations are in agreement with structural predictions (fig. S5), which indicate not only the presence of two conserved calcium ion binding sites but also the absence of the manganese binding site and the surface-exposed tryptophan involved in long-range electron transfer, which are two key features of lignin-active peroxidases (*33*).

Despite its comparatively low peroxidase activity, *Cor*Perox activates *Cor*AlcOx for oxidation of primary alcohols in a dose-dependent manner, and to a much greater extent than the plant peroxidase HRP does (Fig. 2B). The pH optima of *Cor*AlcOx and *Cor*Perox were markedly different (ca. 8 and 4, respectively; fig. S4D). The pH of the environment measured on cucumber cotyledons at the time of triggering appressorium penetration by *C. orbiculare* was between 7.5 and 8.0. Together, these results indicate that AlcOx activation is not dependent on highly efficient peroxidase activity. We also heterologously produced the Perox-AlcOx pair from the rice blast pathogen *M. oryzae*, of which *Mor*AlcOx was recently confirmed to be a primary AlcOx (*34*). Here, we obtained an activation profile for the *Mor*Perox-*Mor*AlcOx pair resembling that observed for the *C. orbiculare* pair (fig. S4E, cf. Fig. 2B).

Having determined optimal enzyme activation conditions, we then probed further the activity of the *Cor*Perox-*Cor*AlcOx pair on biologically relevant aliphatic alcohols. Despite challenges associated with substrate solubility in aqueous buffer, we clearly detected activity on hexadecan-1,16-diol and octadecan-1-ol, as well as on a crude preparation of waxes extracted from cucumber cotyledons (Fig. 2C). This activity was detected only when both enzymes were present. Product analysis by gas chromatography unambiguously indicated that octadecan-1-ol was oxidized to the corresponding aldehyde (fig. S4F).

To obtain a deeper understanding of the activation of AlcOx by the Tandem Peroxidase, we analyzed electron transfer by electron paramagnetic resonance (EPR) spectroscopy (Fig. 2D) and state-of-the art molecular modeling (Fig. 2E and fig. S6). Reduction in the EPR signal of the inactive Cu(II)–nonradical form of *Cor*AlcOx upon addition of *Cor*Perox was supportive of one-electron oxidation leading to the EPR silent, active Cu(II)-radical form (Fig. 2, A and D). This change in electronic structure, observed in the absence of any substrate, confirms the activity-independent activating role of *Cor*Perox. It also indicates a close contact between the enzymes during the activation process, which is concordant with our modeling studies (see below) and with the sigmoidal, titration-like curves observed during activity assays carried out in the presence of substrate (Fig. 2B). Further, *Cor*AlcOx-*Cor*Perox top 5 models predicted by two independent computational techniques, PIPER (*35*) and AlphaFold2-Multimer (*36*), consistently placed *Cor*Perox structures in front of the *Cor*AlcOx active site (fig. S6A). Refinement of these models with the all-atom Monte Carlo (MC) software PELE (Protein Energy Landscape exploration) (*37*), which includes protein small rotations and translations followed by an exhaustive side-chain prediction at the interface, resulted in a clear minimum (fig. S6B) where the heme group of *Cor*Perox is oriented toward the *Cor*AlcOx copper ion (Fig. 2E and fig. S6C). Notably, the binding surfaces of AlcOx orthologs are considerably more hydrophobic than that of GalOx (fig. S7, A and B), which is consistent with the idea that AlcOx may have evolved to interact with the hydrophobic plant cuticle. Moreover, in the top model (Fig. 2E), there is a substantial decrease of solvent exposure of the *Cor*AlcOx active site, defining a cavity between both enzymes that could facilitate diffusion and binding of a long-chain alcohol substrate. C18 docking and PELE induced fit simulations confirm this point, revealing a pronounced local minimum in which the alcoholic group of the substrate is well positioned for catalysis (fig. S6D).

### The Perox-AlcOx pair gates plant penetration

**Fig. 3.**
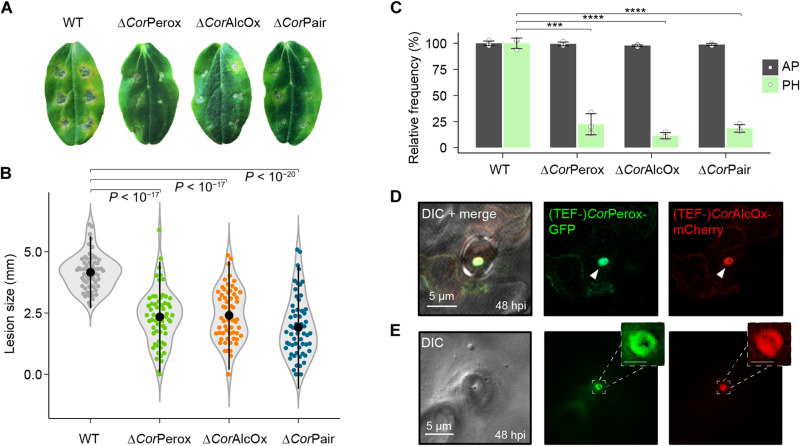
In vivo characterization of the role of the Perox-AlcOx pair during plant infection. (**A** and **B**) Infection phenotypes (A) and violin plot of the necrotic lesion size (B), at 5 dpi, for the wild-type (WT) and *perox/alcox* deletion mutants of *C. orbiculare* on intact cucumber (*C. sativus*) cotyledons. For each strain/plant combination, at least 60 inoculations were carried out. (**C**) Relative frequency (WT set to 100%) of development of normal appressorium (AP) and penetrating hyphae (PH) by *C. orbiculare* strains on cucumber cotyledons. Data are presented as average values (>300 appressoria for each replicate, *n* = 3 independent biological replicates), and error bars show SD. (**D** and **E**) Localization of the *C. orbiculare* tandem oxidases on cotyledons at 48 hpi, in the presence of appressoria cells (D) or after detachment of the appressoria from the surface of the leaf (E). See movie S1 for a 3D view. In (D), white arrowheads indicate enzyme accumulation at the appressorium pore. In (E), enlarged insets show a ring-like localization of the enzymes. Scale bar, 1 μm. In (B) and (C), a one-tailed independent *t* test for each mutant versus WT was applied (***P < 0.001 and ****P < 0.0001).

To investigate the role of the Perox-AlcOx pair in plant infection, we isolated single and double gene deletion mutants of *C. orbiculare* (fig. S8). Inoculation of spore suspensions onto intact cucumber cotyledons showed that fewer and smaller lesions were formed by all the mutants compared to the wild-type strains (Fig. 3, A and B). For instance, the proportion of lesions with a diameter of >4 mm fell from 95% to <20%. Furthermore, similar phenotypes were obtained for single and double mutants, suggesting that both oxidases are crucial for fungal pathogenicity.

Further investigations indicated that neither single nor double *perox*/*alcox* deletion mutants were affected in mycelial growth (fig. S8A). Microscopy revealed that the significant loss of pathogenicity of the mutants was due to a large decrease in the frequency of host penetration (Fig. 3C). However, morphogenesis, cell wall melanization, and turgor buildup within appressoria cells were virtually indistinguishable from those of the wild-type strain (fig. S8, B to D). The next step in the infection process is the emergence of a needle-like penetration peg through a pore in the basal cell wall of the appressorium (Fig. 1A), during which actin assembly at the pore provides rigidity (*38*). Using a red fluorescent protein/actin-binding protein fusion (Lifeact-RFP), we found normal actin assembly at the appressorium pore for both the single and double mutants inoculated onto cucumber cotyledons (fig. S8, E and F).

The mutants could penetrate and form hyphae inside inert cellophane membranes (fig. S9, A to C) and caused wild-type–like lesions when inoculated on mechanically wounded cucumber cotyledons (fig. S9, D and E), in contrast to the crippled invasive capacity observed on intact cotyledons (Fig. 3C). This points to a mechanism involving plant surface compounds, concordant with the catalytic activity of the Perox-AlcOx pair (Fig. 2C). These results collectively indicate that *Cor*AlcOx and *Cor*Perox play a crucial role during the early penetration stage but are not involved in either appressorium or peg formation.

To further examine the function of the Perox-AlcOx pair during plant infection, we attempted to localize the proteins by live-cell imaging of *Cor*AlcOx-mCherry and *Cor*Perox-GFP (green fluorescent protein) driven by their native promoters. Although *Cor*AlcOx-mCherry and *Cor*Perox-GFP complemented the defect in pathogenicity of the deletion mutants, fluorescence of *Cor*AlcOx-mCherry and *Cor*Perox-GFP was not detectable during appressorium formation on cucumber cotyledons (fig. S10A), suggesting that gene expression was too low, consistent with transcriptomic data (Fig. 1C), or that the gene products were secreted and diffused away from the penetration site. However, the constitutive overexpression of *Cor*AlcOx-mCherry and *Cor*Perox-GFP, driven by the translation elongation factor (TEF) promoter, revealed that both proteins accumulated specifically at the appressorial penetration pore and that signal intensity increased during penetration peg formation (Fig. 3D; fig. S10, B and C; and movie S1). This protein colocalization observed in vivo is consistent with gene coexpression data (Fig. 1C) and rationalizes the cooperative activity demonstrated by biochemical assays (Fig. 2). *Cor*AlcOx-mCherry and *Cor*Perox-GFP were detected at the plant surface beneath detached appressoria, at the penetration site (Fig. 3E), suggesting that the tandem metalloenzymes are secreted from appressoria into the plant epidermis. Strikingly, *Cor*AlcOx-mCherry and *Cor*Perox-GFP were not detected at the penetration site on cellophane membranes (fig. S10D), suggesting that interaction of the fungus with the plant surface triggers local and specific recruitment of the tandem metalloenzymes to the pore.

To probe the presence of potential natural substrates of the fungal AlcOx in the neighborhood of the penetration site, we carried out a compositional analysis of waxes present at the surface of uninoculated cucumber cotyledons (fig. S11). This analysis showed that the extracted waxes are mainly composed of odd-numbered alkanes (C27-C33) and even-numbered long-chain primary alcohols (C24-C32). Fatty aldehydes were found only as traces. Thus, this experiment demonstrates that potential substrates of AlcOx represent a major part of the plant cuticular compounds, while AlcOx reaction products are very minor components.

**Fig. 4.**
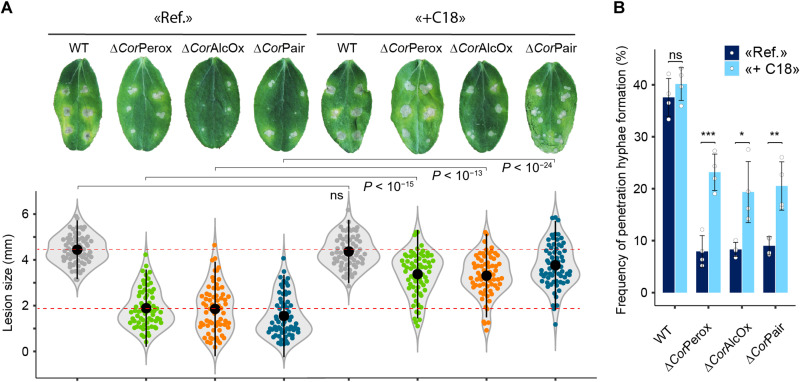
Effect of *n*-octadecanal on the pathogenicity of *perox/alcox* deletion mutants. The figure shows the effect of the addition of 10 μM *n*-octadecanal (denoted as +C18) on necrotic lesion size formed by *C. orbiculare* WT and mutant strains on cucumber cotyledons (24°C, 5 days) (**A**) and on the formation of penetration hyphae (**B**). Control experiments, i.e., 1% ethanol without addition of *n*-octadecanal, are denoted as Ref. In (A), for each strain, we show one representative cotyledon image of the infection phenotype and, below, a violin plot of lesion sizes based on 66 inoculation sites per condition (carried out over four independent biological replicates). In (B), data are presented as mean percentages (>300 appressoria per replicate, *n* = 4 independent biological replicates) and error bars show SD. In (A) and (B), for each strain, a one-tailed independent *t* test of +C18 versus Ref was applied.

To further explore the role of Perox-AlcOx in the fungus-plant dialogue, we exposed the *C. orbiculare* single and double mutants to a product of the AlcOx, viz. the aliphatic long-chain aldehyde *n*-octadecanal. For all gene deletion mutants, the addition of *n*-octadecanal partially restored appressorium penetration ability and lesion formation on cucumber leaves (Fig. 4, A and B), suggesting that the role of the fungal Perox-AlcOx pair is to generate long-chain aldehydes to prime the fungus for efficient plant infection.

Together, our results suggest that the role of the fungal Perox-AlcOx pair is to increase locally the concentration of long-chain aldehydes. These aliphatic compounds, members of the volatile organic compounds, are well known to function as signal molecules (*39*), raising the possibility that the Perox-AlcOx pair generates signals to prime the fungus for efficient plant infection.

**Fig. 5.**
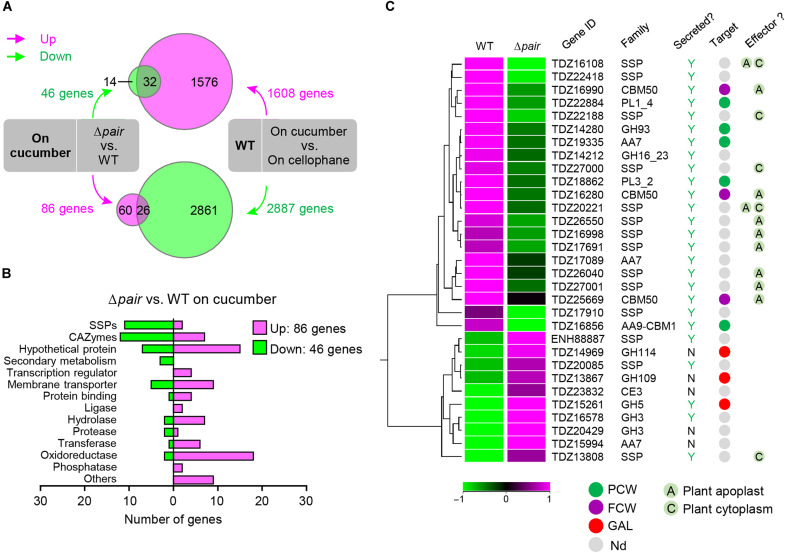
Loss of the Perox-AlcOx pair affects fungal gene expression during appressorium-mediated penetration of *C. orbiculare*. (**A**) Venn diagrams illustrating the differentially regulated (up- or down-regulated) genes between cucumber versus cellophane that are under control of the AlcOx-Perox pair. Top: Green circle indicates the number of down-regulated genes (46 genes) in Δ*pair* compared with wild-type strain (WT), both incubated on cucumber; magenta circle denotes the number of genes (1608 genes) up-regulated on cucumber versus cellophane in the WT strain. Bottom: Magenta circle indicates the number of up-regulated genes (86 genes) in Δ*pair* compared with WT, both incubated on cucumber; green circle shows the number of down-regulated genes (2887 genes) in the WT strain on cucumber versus cellophane. In the top and bottom Venn diagrams, the overlap indicates that 32 and 26 genes are respectively up-regulated or down-regulated in the presence of the plant and also under control of the AlcOx-Perox pair. (**B**) Class-wise distribution of the number of genes that were differentially expressed in appressoria of the Δ*pair* double mutant compared with WT on cucumber cotyledons. (**C**) Hierarchical clustering heatmap of differentially expressed genes encoding CAZymes and SSPs in the Δ*pair* mutant compared to WT on cucumber cotyledon. The expression values per gene were median-normalized. The columns were clustered by Euclidean distance. The expression levels of up-regulated (magenta) and down-regulated (green) genes are shown as log_2_-transformed values. On the right-hand side of the figure, we show the presence of a predicted signal peptide (Y, yes; N, no), phylogeny-based substrate specificity predictions for CAZymes (PCW, plant cell wall; FCW, fungal cell wall; GAL, galactose-containing compounds; Nd, not determined), and SSPs/CBM50s that are putative effectors (pale green circles) predicted to localize in the plant cell [in the apoplast (A) and/or the cytoplasm (C)]. See table S6 for more details.

To gain insights into the possible steps controlled by the Perox/AlcOx activities during the early infection process, we performed comparative transcriptomic analyses of *C. orbiculare* wild-type and double-mutant strains at the appressorial stage on cucumber leaves and cellophane (Fig. 5). Analysis of the differentially expressed genes suggested that the Perox-AlcOx pair contributes to the regulation of a subset of 32 plant-inducible genes predicted to encode small secreted proteins (SSPs), carbohydrate-active enzymes (CAZymes), and membrane transporters (table S6). SSPs and CAZymes are well-known fungal effectors playing a key role in the molecular dialog with host plants. A phylogenetic analysis of CAZymes (table S6) present in this subset of genes revealed that the pair is required for the up-regulation of genes encoding proteins directed toward the plant cell wall (PCW) and the fungal cell wall (FCW) (Fig. 5). Among the former, we detected cellulose-active enzymes known to display an enzymatic interplay—viz. the cello-oligosaccharide dehydrogenase AA7 and the lytic polysaccharide monooxygenase (LPMO) AA9 (*40*)—as well as pectin-active enzymes from the PL1, PL3, and GH93 CAZy families. Regarding the FCW-targeting proteins, we detected three proteins with carbohydrate-binding modules (CBM50s; also called LysM domains) that bind to chitin and function to evade recognition by host immune receptors during infection (*41*).

## DISCUSSION

**Fig. 6.**
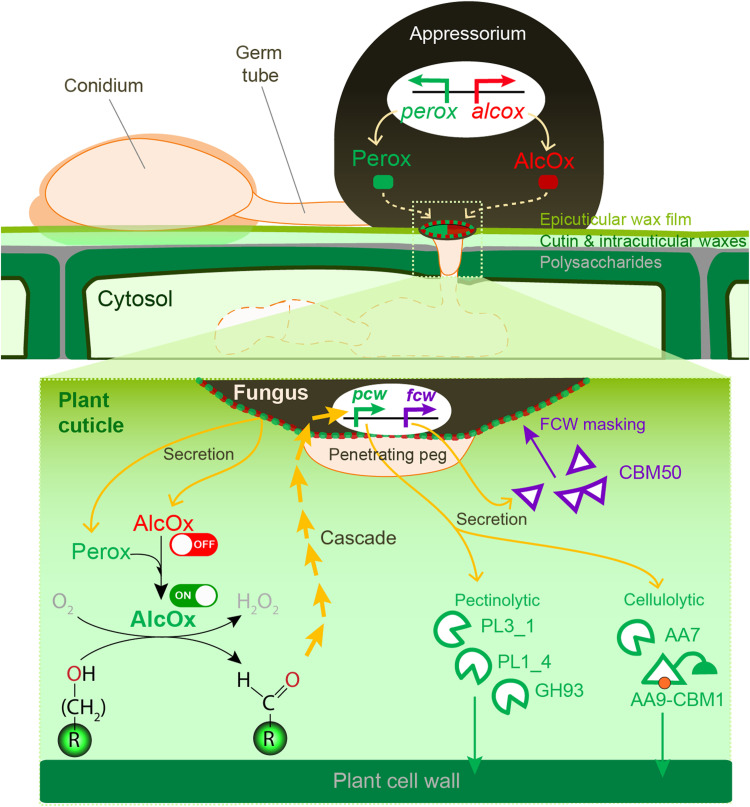
Schematic summary of the recruitment of the fungal Perox-AlcOx pair during early plant penetration and its proposed role in the induction of a biochemical cascade. The bottom panel is a zoom-in view illustrating the Perox-AlcOx reaction occurring at the fungus-plant interface and the triggered downstream cascade, highlighting the induction of genes encoding proteins targeting the plant cell wall (*pcw*) and fungal cell wall (*fcw*).

Research on plant invasion by appressorium-forming fungi teaches us that the development of these specialized infection structures is an extremely complex, finely regulated process. In this study, we have shown that fungal AlcOx, which are only encountered in phytopathogens, have a very different biological function than the few distantly related CROs for which roles in morphogenesis (*42*, *43*) or lignin degradation (*18*) were proposed. We showed that the tandem Perox-AlcOx metalloenzymes are specifically deployed at the initiation of penetration peg formation and their localization at the pore formed at the fungus–plant cell interface. Our results suggest that the oxidative action of Perox-AlcOx on long-chain alcohols triggers a yet-to-be-elucidated biochemical cascade leading to penetration. Functional complementation of the deletion mutants, which were defective in their ability to puncture intact plant cuticles, by the product of the reaction suggests that cuticular alcohol oxidation provides a chemical cue required for plant cell entry (Fig. 6). We speculate that acquisition of the Perox-AlcOx pair provided the ecologically widespread *Colletotrichum* and *Magnaporthe* species with an advantage, namely, an in-house “locksmith” (i.e., the tandem metalloenzymes) ensuring localized production of the aldehyde. This entry key is needed to move to the next pathogenesis stage with the expression of PCW-degrading enzymes to facilitate penetration, and of FCW-binding proteins and carbohydrate oxidases to evade host immunity. For example, it has been suggested that, upon oxidation by fungal LPMOs and oligosaccharide oxidases, plant oligosaccharides can no longer play their role as inducers of host immune response (*40*, *44*, *45*).

It is interesting to speculate why nature has evolved such a complex enzymatic system relying on heme iron peroxidase and elaborate copper radical chemistry when simpler, mono-enzyme systems could be used for the same purpose [e.g., FAD-dependent oxidases active on long-chain alcohols (*46*)]. We show that the functional integrity of the secreted tandem metalloenzymes system endows pathogenic fungi with fine control over oxidation reactions in the extracellular space. We propose that the copper radical center equips CROs with a “redox switch” to turn on activity with high spatiotemporal resolution, when paired with a cognate peroxidase (Fig. 6). More generally, our study suggests that fungi have evolved ways of controlling oxidative reactions that are seemingly out of reach, i.e., beyond the bounds of the fungal plasma membrane and cell wall and through tight genetic regulation and protein interplay between secreted oxidoreductases.

In conclusion, using a combination of in silico, in vitro, and in vivo approaches, we have unveiled the existence of a natural redox partner, i.e., a heme-peroxidase, for AlcOx-type CROs and showed that the pair acts as a secreted virulence factor during early infection (Fig. 6). It is noteworthy that the enzymology-driven approach pursued here was essential to bring to light this unique mechanism, because low expression levels, fine temporal tuning, and highly localized cosecretion of the Perox-AlcOx would have evaded classical ‘omic approaches. The specific occurrence of the Perox-AlcOx pair in most *Colletotrichum* and *Magnaporthe* species raises the possibility that functionally equivalent, coupled oxidative enzymatic mechanisms may operate in other appressorium-forming fungal pathogens. We anticipate that the present discovery will open new research avenues, notably on the role of wax-derived compounds in the cascade of events leading to successful infection as well as on the development of oxidase-specific inhibitors as surface-acting, anti-penetrant drugs for crop protection.

## MATERIALS AND METHODS

### Chemicals and commercial enzymes

Most chemicals were purchased from Sigma-Aldrich (Darmstadt, Germany) or VWR (Fontenay-sous-Bois, France) unless stated otherwise. Molar concentrations of type II HRP (Sigma-Aldrich; *M*_w_, 33.89 kDa) were estimated by Bradford assay. *n*-Octadecanal was purchased from TCI-Europe (Zwijndrecht, Belgium). All alcohol substrate stock solutions were prepared either in H_2_O or in acetone and stored at −20°C. The concentration of H_2_O_2_ stock solution was verified at 240 nm (ε^240^ = 43.6 M^−1^ cm^−1^).

### Bioinformatics

Species tree of the 18 ascomycete genomes (shown in Fig. 1B) was constructed as previously described (*47*). Core clusters containing only one protein-coding gene per species were identified using FastOrtho (*48*) with the following parameters: 50% identity and 50% coverage. Each cluster was aligned with MAFFT (Multiple Alignment using Fast Fourier Transform) 7.221 (*49*), and curated alignments were concatenated with Gblocks 0.91b (*50*). The tree was finally constructed with RAxML 7.7.2 (*51*) (PROTGAMMAWAG model and 500 bootstrap). Phylogenetic analysis of AA5_2 genes and «standard» haem peroxidases (PFAM 00141, including class II peroxidases) from 30 *Colletotrichum* genomes relied on 72 and 333 sequences, respectively (see fig. S2 legend and table S3 for more details). The phylogenetic analysis of the peroxidase-catalase superfamily relied on 150 sequences encompassing class I (intracellular peroxidases), class II (fungal secreted peroxidases), and class III (plant secreted peroxidases) peroxidases and from so-called hybrid B peroxidases, as previously reported (*52*). Subsequently to manual curation (removing signal peptides), the sequences were first aligned using MAFFT-DASH (L-INS-i method) (*53*) and the resulting multiple sequence alignment (MSA) was used to infer a phylogenetic tree using RAxML (1000 bootstraps). The trees were then visualized in iTOL (*54*) and edited in Illustrator.

For the gene neighborhood survey, we retrieved four genes located upstream and downstream of each *Colletotrichum*’s AA5_2 query (72 sequences). The resulting 569 genes were assigned to 78 different PFAM domains. The frequency of occurrence of a given type of domain in the neighborhood of each AA5_2 phylogenetic clade was then computed and visualized in Excel.

For interrogating the cooccurrence of both Perox and AlcOx-coding genes beyond *Colletotrichum* species, two independent BLAST searches were run against the National Center for Biotechnology Information (NCBI) nonredundant database, using *Cor*AlcOx and *Cor*Perox as query sequences. One thousand AlcOx-like (down to 37% sequence identity) and 1000 Perox-like (25% sequence identity) sequences along with their corresponding source microorganism were retrieved. A cross-comparison of both lists of microorganisms, applying different sequence identity-based thresholds (60% for AlcOx and 30% for Perox), returned the list of species harboring both type of enzymes.

Transcriptomics data were retrieved from publicly available datasets (*26*–*29*). To normalize different dataset longitudinally, we expressed reported sampling time points as infection stages in Fig. 1 as follows (hpi, hours post-infection): for *Ciboria fructicola nara gc5*/strawberry (*27*): AP (24 hpi), BP (72 hpi), and NP (144 hpi); for *C. orbiculare*/*Nicotiana benthamiana* (*27*): AP (24 hpi), BP (72 hpi), and NP (168 hpi); for *Colletotrichum higginsianum*/*Arabidopsis thaliana* (*26*): AP (22 hpi), BP (40 hpi), and NP (60 hpi); *C. graminicola*/*Maize* (*26*): AP (22 hpi), BP (40 hpi), and NP (60 hpi); *M. oryzae*/*Rice* (*28*), AP (8 hpi), BP (24 hpi), and NP (48 hpi); *M. oryzae*/*Barley* (*29*): AP (12 hpi), PP (24 hpi), BP (36 hpi), and NP (48 hpi).

### Structure prediction and preparation

#### Surface hydrophobicity

Structural homology models were generated with AlphaFold (*55*), and surface hydrophobicity of selected CROs was computed with the “protein-sol patches” online software (*56*). Average hydrophobicity of the binding surface was determined as follows: Using PyMOL 2.4, we selected the residues constituting the entire binding surface of *Fgr*GalOx, *Cgr*AlcOx, and equivalent residues (based on MSA) in orthologous enzymes (5 GalOx and 11 AlcOx in total) as well as those of four characterized hydrophobins (PDB 2N4O, 2LSH, 1R2M, and 2FZ6) as hydrophobic protein reference. Average hydrophobicity of the selected residues was computed as a GRAVY index score (Kyte-Doolittle method) (*57*).

#### Preparation of models for docking experiments

We used Alphafold2 (*55*) to obtain a model for *Cor*AlcOx and *Cor*PerOx. To add the metals and cofactors to AlphaFold2 models, we performed a BLAST search in the Protein Data Bank protein database and selected the PDB 2EIC (Sequence Identity 47%) to add the copper to *Cor*AlcOx and the PDB 1MN2 (Sequence Identity 28%) to add the calcium ions and the heme group to *Cor*Perox. Subsequently, we prepared the systems with Schrödinger Protein Preparation Wizard (*58*) to determine the protonation states at pH 7 using PROPKA (*59*) and finally relax the systems performing a restrained minimization with convergence criteria for heavy atoms to 0.30 Å using the OPLS_2005 force field.

#### Protein-protein docking

For the generation of protein-protein poses (PPPs), we used PIPER (*35*) to generate 70,000 PPPs, which were clustered with the in-built clustering protocol using the default root mean square deviation threshold of 9.0 Å and a minimum population of 10 poses per cluster. In parallel, we also used the recently developed Alphafold2 Multimer (*36*), which only uses sequence information, together with ion and cofactor placements, as described above.

#### PPP refinement with PELE

We used the all-atom MC software PELE to map intermolecular interactions (*37*, *60*). PELE follows a heuristic MC approach, generating new conformational proposals using vibration modes of the proteins with translations and rotations of the ligand (*Cor*Perox in this case), and relaxing the system with structure prediction methods, so that the probability of acceptance in the Metropolis criterion (*61*) remains high (*60*). Here, we refined the top five PIPER and five AlphaFold2-Multimer models, using a simulation of 250 PELE steps with 256 computing cores (about 25 independent trajectories per model). Each MC PELE step consists of a perturbation and a relaxation stage. In the first one, a perturbation of the ligand, *Cor*AlcOx in this case, is first performed, including random rotations of 0.01 to 0.04 rad and translations of 0.25 to 0.50 Å, and is followed by a backbone perturbation of both proteins following the normal mode directions predicted by an Anisotropic Network Model (ANM). In the second stage, the system is first relaxed by a high-resolution side-chain prediction including all protein-protein interphase side chains, defined by the region within 3 Å of any heavy atom of the other protein. Afterward, a global minimization is performed to relax the entire system, providing a final conformation and energy; this energy is then used in a Metropolis importance sampling to accept/reject the MC step.

#### Exploring the substrate interactions

The C18 substrate was created with Maestro 3D Builder, followed by a Glide docking (*62*) on the lowest-energy PPPs, as predicted with PELE. The docking grid was defined with a cubic box of 30 Å centered in between the copper ion and Tyr^120^(OH) of the *Cor*AlcOx. Following the Glide rigid receptor docking, we performed a C18-induced fit rescoring simulation involving 40 PELE steps (using 48 computing cores). Each PELE step consisted of a random rotation of 0.01 to 0.04 rad and translation of 0.05 to 0.15 Å, using a spherically restrained search space of radius 12 Å centered on the copper ion. The perturbation step was then followed by a relaxation phase including side-chain prediction (all side chains inside the spherical space) and a full system minimization.

### DNA cloning and strain production

DNA cloning and strain production of the AA5_2 AlcOx from *Colletotrichum graminearum* (*Cgr*AlcOx, GenBank ID XM_008096275.1, UniProt ID E3QHV8) were already carried out in previous study (*19*). The intron-free sequences of the genes coding for the AlcOx from *C. orbiculare* MAFF 240422 (*Cor*AlcOx, GenBank ID TDZ17043.1, UniProt ID N4UTF2), the AlcOx from *M. oryzae* (*Mor*AlcOx, GenBank ID XM_003719321.1, UniProt ID G4NG45), the Tandem Peroxidase (Perox) from *C. orbiculare* (*Cor*Perox, GenBank ID TDZ17044.1, UniProt ID N4UUY4), and the Tandem Peroxidase from *M. oryzae* (*Mor*Perox, GenBank ID XM_003719322.1, UniProt ID G4NG46) were synthesized after codon optimization for expression in *P. pastoris* and inserted into a modified pPICZαC vector using Xho I* and Not I restriction sites in frame with the α secretion factor at the N terminus (i.e., without native signal peptide) and with a (His)_6_-tag at the C terminus (without *c*-myc epitope) (Genewiz, Leipzig, Germany). Transformation of competent *P. pastoris* X33 and selection of zeocin-resistant *P. pastoris* transformants screened for protein production were carried out as described by Haon *et al.* (*63*). The best-producing transformants were conserved as glycerol stock at –80°C.

### Heterologous protein production in flasks

All proteins were first produced in 2-liter Erlenmeyer flasks. To this end, single colonies of *P. pastoris* X33 expressing each gene of interest were individually streaked on a YPD (yeast extract, peptone, and dextrose) agar plate containing zeocin (100 μg ml^−1^) and incubated for 3 days at 30°C. A single colony was then used to inoculate 5 ml of YPD, in a 50-ml sterile Falcon tube, and incubated during 5 hours (30°C, 160 rpm). This preculture was used to inoculate at 0.2% (v/v) 500 ml of BMGY (buffered glycerol complex medium), in a 2-liter Erlenmeyer flask, and incubated during approximately 16 hours (30°C, 200 rpm) until OD_600_ (optical density at 600 nm) reached 4 to 6. The produced cellular biomass was then harvested by centrifugation (5 min, 16°C, 3000*g*). For the AlcOx, the cell pellet was then resuspended in 100 ml of BMMY (buffered methanol complex medium) supplemented with methanol (1%, v/v) and CuSO_4_ (500 μM). The culture was incubated for 3 days (16°C, 200 rpm), with daily additions of methanol (1% added, v/v). The Tandem Peroxidase production conditions were optimized and varied from the standard protocol as follows: The BMMY was supplemented with methanol (3% v/v), hemin (25 μM), and CaCl_2_ (2 mM). The culture was incubated for 3 days (20°C, 200 rpm), with daily additions of methanol (3%, v/v) and hemin (25 μM). Then, the extracellular medium was recovered by centrifugation (10 min, 4°C, 3000*g*), and the supernatant was filtrated on 0.45-μm membrane (Millipore, Massachusetts, USA) and stored at 4°C before purification.

### Heterologous protein production in bioreactors

The upscaled production of *Cor*Perox was carried out in 1.3- and 7.5-liter bioreactors (New Brunswick BioFlo 115 fermentor, Eppendorf, Germany) as per the *P. pastoris* fermentation process guidelines (Invitrogen) with the following optimizations: The glycerol fed-batch phase was replaced by a sorbitol and methanol transition phase; besides, 200 μM (1.3-liter bioreactor) and 150 μM (7.5-liter bioreactor) of hemin were added to the methanol solution. CaCl_2_ (10 mM final) was added to the crude protein solution before being either directly purified or flash-frozen in liquid nitrogen and stored at −80°C. We verified that flash-freezing did not cause any activity loss, for both AlcOx and Perox enzymes.

### Protein purification

The filtered *Cor*AlcOx and *Mor*AlcOx crude supernatants were adjusted to pH 8.5, filtered on 0.22-μm filters (Millipore, Molsheim, France), and purified by anion exchange chromatography (DEAE) on a HiPrep FF 16/10 column (GE Healthcare, USA). Elution was performed by applying a linear gradient from 0 to 500 mM NaCl (in tris-HCl buffer 50 mM, pH 8.5) over 20 column volumes, with a flow rate set to 5 ml min^−1^.

The filtered *Cor*Perox and *Mor*Perox culture supernatant was adjusted to pH 7.8 just before purification and filtered on 0.22-μm filters (Millipore, Molsheim, France). Depending on the volume to purify, the crude protein sample was loaded on a His-Trap HP 5-ml column (GE Healthcare, Buc, France) on a HisPrep FF 16/10 column (GE Healthcare) connected to an ÄKTAxpress system (GE Healthcare) equilibrated with Hepes (10 mM, pH 8.0), NaCl (100 mM), CaCl_2_ (2 mM), and imidazole (10 mM) buffer. Each (His)_6_-tagged recombinant enzyme was eluted with Hepes (10 mM, pH 8.0), NaCl (100 mM), CaCl_2_ (2 mM), and imidazole (500 mM) buffer. The Tandem Peroxidases were further purified by size exclusion chromatography, using a HiLoad 26/600 Superdex 200 pg column (GE Healthcare) operated at 2.5 ml/min and with a running buffer containing Hepes (10 mM, pH 8.0), NaCl (100 mM), and CaCl_2_ (2 mM).

After SDS-PAGE analysis, fractions containing the recombinant enzyme were pooled, concentrated, and buffer-exchanged in sodium phosphate (50 mM, pH 7.0) for the AlcOx or in Hepes (10 mM, pH 8.0), NaCl (100 mM), and CaCl_2_ (2 mM) buffer for the Tandem Peroxidases.

Protein concentrations of *Cgr*AlcOx (52,337 Da, ε^280^ = 101,215 M^−1^ cm^−1^), *Cor*AlcOx (52,317 Da, ε^280^ = 92,735 M^−1^ cm^−1^), *Mor*AlcOx (62,894 Da, ε^280^ = 90,020 M^−1^ cm^−1^), *Cor*Perox (26,137 Da, ε^280^ = 21,345 M^−1^ cm^−1^), and *Mor*Perox (26,290 Da, ε^280^ = 24,450 M^−1^ cm^−1^) were determined by the Bradford assay (*64*) using bovine serum albumin as reference protein as well as by ultraviolet (UV) absorption at 280 nm using a NanoDrop ND-200 spectrophotometer (Thermo Fisher Scientific, Massachusetts, USA).

### Enzyme assays

For screening the substrate specificity of CRO-AlcOx enzymes, the alcohol substrates were prepared in sodium phosphate buffer (50 mM, pH 7.0) in 96-well microplates and reactions were initiated by the addition of a premix of CRO-AlcOx (1 nM final concentration), HRP (0.1 mg ml^−1^), and 2,2′-azino-bis(3-ethylbenzothiazoline-6-sulfonic acid (ABTS; 500 μM) in sodium phosphate buffer (50 mM, pH 7.0). The tested substrates included d-glucose (50 mM final concentration), d-galactose (50 mM), d-raffinose (50 mM), xyloglucan (0.1% mM), butan-1-ol (3 mM), butan-2-ol (3 mM), octan-1-ol (3 mM), decan-1-ol (3 mM), 2,4-hexadiene-1-ol (3 mM), glycol aldehyde dimer (3 mM), benzyl alcohol (3 mM), 4-hydroxybenzyl alcohol (3 mM), vanillic alcohol (3 mM), syringic alcohol (3 mM), and cinnamyl alcohol (3 mM). The absorbance of the final reaction (100-μl total volume) was monitored at 414 nm using a microplate spectrophotometer (TECAN) and thermostated at 23°C. The 414-nm absorbance allows to determine the concentration of ABTS cation radical over time (ABTS^•+^, ε^414^ = 31,100 M^−1^ cm^−1^) and, in turn, the rate of alcohol oxidation, considering a peroxidase reaction stoichiometry for (H_2_O_2_:ABTS^•+^) of 1:2 and a CRO-AlcOx reaction stoichiometry for (alcohol:H_2_O_2_) of 1:1.

For screening the substrate specificity of *Cor*Perox, unless stated otherwise, the enzyme (0.125 μM final) was prepared in citrate-phosphate buffer (50 mM, pH 4.0 to 7.0) in 96-well microplates (for wavelength in the visible range) or in 1-ml Quartz cuvettes (for UV range), in the presence of various substrates (vide infra). Reactions were initiated by the addition of H_2_O_2_ (100 μM final), incubated at 23°C, and monitored spectrophotometrically at the wavelengths indicated below. The tested peroxidase substrates included the following: ABTS (500 μM) converted into ABTS^•+^ (ε^414^ = 31,100 M cm^−1^), 2,6-dimethoxyphenol (500 μM) converted into hydrocoerulignone (ε^469^ = 53,200 M cm^−1^), guaiacol (500 μM) converted into the final product tetraguaiacol (ε^470^ = 26,600 M cm^−1^), Reactive Black 5 (RB5; 100 μM, ε^600^ = 20,000 M cm^−1^) converted into nonchromogenic product RB5^ox^, and veratryl alcohol (500 μM) converted into veratraldehyde (ε^310^ = 9300 M cm^−1^). For testing the manganese peroxidase activity, *Cor*Perox was mixed with Mn(II)SO_4_ (1 mM final) in tartrate buffer (50 mM, pH 2.0 to 5.0) and the formation of Mn^3+^-tartrate complex upon addition of H_2_O_2_ (100 μM) was followed at 238 nm (ε^238^ = 6500 M^−1^ cm^−1^), as previously described (*65*).

All activities were expressed as Vi/E (s^−1^), i.e., the initial rate (Vi, μmol of H_2_O_2_ consumed per second) divided by the amount of enzyme (in μmol). *Cor*Perox stability over time was carried out by monitoring the peroxidase activity of *Cor*Perox samples (50 μM) stored in sodium acetate buffer (50 mM, pH 5.2), at 4°C, in the presence of varying concentrations of CaCl_2_ (0 to 500 mM). The peroxidase activity of these samples was measured as described above (final concentration of 0.5 μM *Cor*Perox), using ABTS (500 μM) and H_2_O_2_ (100 μM) as substrates, in citrate-phosphate buffer (50 mM, pH 4.0), at 23°C.

Michaelis-Menten kinetic parameters of *Cor*Perox were determined by measuring the peroxidase initial rate, as described above, in the presence of ABTS (500 μM) and varying concentrations of H_2_O_2_ (0 to 1200 μM), in citrate-phosphate buffer (50 mM, pH 4.0), at 23°C. Experimental data could be fit to the standard Michaelis-Menten equation (residual standard error = 0.019).

AlcOx activation by the peroxidases was assayed by monitoring changes in absorbance at 254 nm upon oxidation of benzyl alcohol (1.5 mM) into benzaldehyde by the CRO (10 nM final concentration), in the presence of varying concentrations of peroxidase (0 to 1000 nM). Reactions were carried out in sodium-phosphate buffer (50 mM, pH 7.0), at 23°C, in UV-transparent cuvettes (1 ml reaction volume). The reactions were initiated by addition of the CRO and vigorously mixed by pipetting up and down. The absorbance was measured using an Evolution 201 UV-Vis spectrophotometer (Thermo Fisher Scientific). The concentration of benzaldehyde was calculated as [Benzaldehyde]_t_ = (Abs^254 nm^_t_ − Abs^254 nm^_t0_)/(ε^254^_benzaldehyde_ − ε^254^_BnOH_), where ε^254^_benzaldehyde_ = 8500 M cm^−1^ and ε^254^_BnOH_ = 150 M cm^−1^.

### Gas chromatography analysis

Enzymatic reactions were carried out in 4-ml clear borosilicate glass vials closed by screw caps with PTFE (Polyethylfluoroethylene) septum (500-μl final reaction volume). *Cor*AlcOx (2 μM final) was mixed with *Cor*Perox (2 μM) in sodium phosphate buffer (50 mM, pH 7.0). The reaction was initiated by the addition of octadecanol (0.3 mg ml^−1^, eq. 1.1 mM final), and the mixture was incubated at 23°C at 190 rpm in an Innova 42R incubator (New Brunswick, USA), during 1 hour. Following a previously published protocol (*22*), the reaction mixture was then acidified by addition of 10 μl of HCl (12 M). Products and possible remaining substrate were extracted by adding 500 μl of hexane (containing 1 mM of internal standard dodecane), followed by shaking and centrifugation for 5 min at 3000*g*. The organic layer was transferred into a new vial and analyzed with a GC-2010 Plus apparatus (Shimadzu, Japan) equipped with a flame ionization detector and a DB-5 capillary column (30 m × 0.25 mm × 0.25 μm; Agilent). Nitrogen (200 kPa) was used as carrier gas. The injector and detector temperatures were set at 250°C. After injection (2-μl sample), the analytes were separated by applying the following temperature program: step 1, from 65° to 250°C over 9.25 min (i.e., 20°C/min); step 2, plateau at 250°C for 6 min. For quantitation, standard curves of octadecanol, *n*-octadecanal, and octadecanoic acid were prepared by following the same procedure.

### Electron paramagnetic resonance

EPR spectra were recorded on frozen solutions (120 K) using a Bruker Elexsys E500 spectrometer operating at X-band equipped with a BVT 3000 digital temperature controller. The following acquisition parameters were used: modulation frequency, 100 kHz; modulation amplitude, 5 G; gain, 87 dB; microwave power, 20 mW. EPR spectra were simulated using the EasySpin toolbox developed for Matlab (*66*). *Cor*AlcOx (100 μM final), prepared in sodium phosphate buffer (50 mM, pH 7.0), in the absence or presence of *Cor*Perox (100 μM final), was flash-frozen in liquid nitrogen, and continuous-wave EPR spectra were recorded. *Cor*AlcOx and *Cor*Perox were placed in contact for various amounts of time (2.5 and 15 min) before flash-freezing the solution. Controls containing buffer only or *Cor*Perox were also carried out.

### Analysis of cuticular waxes from cucumber cotyledons

Cuticular waxes were extracted from 2-week-old cotyledons by immersing six intact cotyledons for 30 s in chloroform in a glass beaker. Chloroform was evaporated under a stream of nitrogen gas, and wax extracts were derivatized using *N*,*O*-bis (trimethylsilyl)trifluoroacetamide and analyzed by gas chromatography coupled to mass spectrometry as previously described (*67*).

### Strains and media

Strain 104-T (MAFF240422) of *C. orbiculare* was used as the wild-type strain. All strains used in this study are listed in table S4 (*13*, *38*, *68*–*70*). *C. orbiculare* strains were cultured on 3.9% PDA (Potato dextose agar) (Nissui) at 24°C in darkness. For genetic manipulation, *Escherichia coli* DH5α-competent cells were maintained on LB agar at 37°C. For fungal transformation, *Agrobacterium tumefaciens* C58C1 was maintained on LB agar at 28°C. Transformations of *C. orbiculare* (*13*, *38*) were carried out as previously described.

### Strain construction

Primers and plasmids used in this study are listed in table S5. For construction of *CorAlcOx* deletion strains, 1.1-kb upstream and 1.0-kb downstream flanking sequences and a 1.0-kb fragment of the neomycin-resistance cassette were amplified with the respective primer pairs. For construction of *CorPerox* deletion strains, 1.1-kb upstream and downstream flanking sequences and a 1.4-kb fragment of the hygromycin-resistance cassette were amplified. These three fragments were inserted into linearized pPZP-PvuII using an In-Fusion HD cloning kit (Clontech). The same procedures were used for construction of *M. oryzae* gene deletion strains.

For construction of *CorAlcOx-mCherry* and *CorPerox-GFP* gene fusion, a 5.9-kb *CorAlcOx-CorPerox* fragment containing 1.1-kb downstream flanking sequences was inserted into linearized pPZP-PvuII-SUR, and the mCherry and GFP fragments were inserted.

For construction of *CorAlcOx-mCherry* overexpression strains, a 4.0-kb *CorAlcOx-mCherry* fragment containing its 1.1-kb downstream flanking sequence was amplified from pPZP-AlcOx-mCherry-Perox-GFP-S and fused to linearized pCAMSUR-TEF (*71*) containing the TEF promoter of *Aureobasidium pullulans* (*72*). The same procedures were used for construction of *CorPerox-GFP* overexpression strains.

### Plant infection

Infection assays on detached cucumber leaves (*Cucumis sativus* L. “Suyo”) with conidial suspension (1 × 10^5^ conidia/ml in distilled water) of *C. orbiculare* were performed as previously described (*73*). The inoculated leaves were incubated in a humid environment for 5 days at 24°C. For testing the effect of *n*-octadecanal supplementation, conidial suspensions (1 × 10^5^ conidia/ml in 10 μM *n*-octadecanal dissolved in 1% ethanol or 1% ethanol as a control) were spotted onto detached cucumber leaves and incubated in a humid environment for 5 days at 24°C.

### Microscopy

For observation of appressorium formation of *C. orbiculare*, a conidial suspension (5 × 10^5^ conidia/ml) was placed on a multiwell glass slide or cover glass (Matsunami Glass), respectively. Cells were incubated in a humid box for 24 hours at 24°C in the dark. Observation of penetration hyphae on cucumber cotyledons and cellophane membranes was performed as previously described (*13*). Appressorial cytorrhysis assay was conducted on the basis of a previous procedure (*68*).

A confocal laser scanning microscope (LSM900) with Airyscan 2 (Carl Zeiss) equipped with a Plan Apochromat 63×/1.4 Oil differential interference contrast objective (Carl Zeiss) was used to acquire confocal microscopic images. Excitation/emission wavelengths were 488 nm/490 to 556 nm for GFP and 561 nm/565 to 630 nm for mCherry. Images were acquired and processed using ZEN Software (version 3.1; Carl Zeiss) and Imaris (version 9.3.1; Bitplane). For detection of appressorial actin assembly, cells were observed using a Zeiss Axio Imager M2 Upright microscope (Carl Zeiss) equipped with a Plan Apochromat 100× oil immersion lens, an Axio Cam MRm digital camera, and excitation/barrier filter set of 595 nm/620 nm for red fluorescent protein (RFP). Images were acquired using Axiovision 4.8. Bright-field microscopy was performed using a Nikon ECLIPSE E600 microscope equipped with a 40× water immersion lens (Nikon) and an OLYMPUS DP74 digital camera system.

### Microarray analysis

For sampling appressoria, the abaxial surface of cucumber cotyledons or cellophane membranes (Wako Chemicals) were inoculated with 10-μl droplets or 10 ml, respectively, of a conidial suspension (1 × 10^6^ conidia/ml) and then incubated at 24°C in a humid box. After 16 hours, the cellophane was frozen in liquid nitrogen. After 24 hours, the lower epidermis of the cotyledons was peeled off. All samples were ground in liquid nitrogen, and total RNA was prepared using the Maxwell RSC Plant RNA Kit (Promega) and the Agilent Plant RNA Isolation Mini Kit (Agilent Technologies). Microarray analyses were performed as described previously (*13*) using the *C. orbiculare* (8 × 60,000, 13,352 independent probes, Design ID: 060762) oligo microarray, according to the Agilent 60-mer Oligo Microarray Processing Protocol (Agilent Technologies). The normalization condition were as follows: (i) intensity-dependent Lowess normalization; (ii) data transformation, measurements less than 0.01 were set to 0.01; (iii) per-chip 75th-percentile normalization of each array; and (iv) the expression values per gene were median-normalized. The normalized data were subjected to a *t* test, with statistically significant gene sets defined as those giving *P* values less than 0.05. The differentially regulated genes (fold change > 2 and *P* < 0.05) were selected and used for further analysis. Functional classification was based on the Gene Ontology (GO), protein families (Pfam), and *C. orbiculare* genome information of CAZymes and SSPs. Note that to avoid redundancy in the count of up- and down-regulated genes, all sequences considered as CAZymes were not counted in the broader classes of hydrolases/transferases/oxidoreductases.

## Supplementary Materials

### This PDF file includes:

Figs. S1 to S11 Tables S1 to S6 References

## Notes

View/request a protocol for this paper from *Bio-protocol*.
