# Supplementary material for: Tandem metalloenzymes gate plant cell entry by pathogenic fungi
Source: Sci Adv. 2022 Dec 21;8(51):eade9982. doi: 10.1126/sciadv.ade9982 (PMC9770985; doi:10.1126/sciadv.ade9982)
Supplement: Supplementary file 1 — Figs. S1 to S11 Tables S1 to S6 References [file sciadv.ade9982_sm.pdf]

Science Advances
AAAS

Supplementary Materials for

Tandem metalloenzymes gate plant cell entry by pathogenic fungi

Bastien Bissaro *et al.*

Corresponding author: Yasuyuki Kubo, yasuyuki.kubo@setsunan.ac.jp; Jean-Guy Berrin, jean-guy.berrin@inrae.fr

Sci. Adv. **8**, eade9982 (2022)
DOI: 10.1126/sciadv.ade9982

The PDF file includes:

Figs. S1 to S11
Tables S1 to S6
Legend for movie S1
References

Other Supplementary Material for this manuscript includes the following:

Movie S1

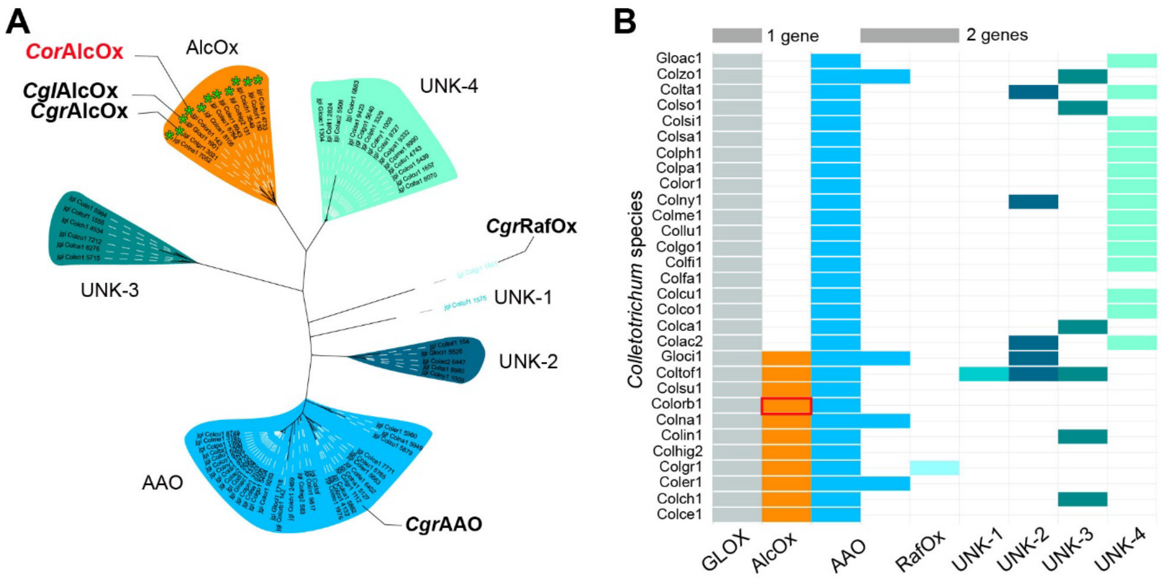

C

Neighborhood

PFAM domains	Description	AlcOx	AAO	RafOx	UNK-1	UNK-2	UNK-3	UNK-4
PF02826	2-hydroxyacid dehydrogenase	0	0	0	0	0	0	87
PF04547	Anoctamin	0	42	0	0	0	0	0
PF05730	CFEM	9	0	0	100	0	0	0
PF01266	D-amino acid oxidase	0	0	0	0	0	33	0
PF09118	DUF1929 (GalOx related)	0	6	0	0	0	33	27
PF12013	DUF3505 (Orsellinic acid biosynthesis)	0	0	0	100	0	0	0
PF12359	DUF3645	55	0	0	0	0	0	0
PF01070	FMN-dependent dehydrogenase	0	79	0	0	0	0	0
PF04828	GFA	45	0	0	0	0	0	0
PF13632	GT2	0	0	0	0	0	67	0
PF07940	Heparinase II/III-like protein	0	0	0	0	0	67	0
PF06985	Heterokaryon incompatibility protein (HET)	9	0	0	0	0	0	87
PF01344	Kelch_1	27	3	0	0	25	50	0
PF07646	Kelch_2	73	0	0	0	0	0	0
PF13964	Kelch_6	0	0	0	100	0	0	0
PF00149	Calcineurin-like phosphoesterase	0	30	0	0	0	0	0
PF07690	Major Facilitator Superfamily 1	64	0	0	0	0	33	0
PF05653	Magnesium transporter NIPA	0	0	0	0	0	0	53
PF03169	OPT oligopeptide transporter protein	0	0	0	0	0	0	87
PF00024	PAN_1	0	91	0	0	25	0	0
PF14295	PAN_4	0	0	100	0	50	0	0
PF00141	Haem peroxidase	100	0	0	0	0	0	0
PF01522	Polysaccharide deacetylase	45	0	0	0	0	0	0
PF10257	Retinoic acid induced 16-like protein	0	0	0	0	0	50	0
PF08477	Ras of Complex, Roc, domain of DAPkinase	0	73	0	0	0	0	0
PF00106	SDR : Short chain Dehydrogenase Reductase	9	3	0	0	0	0	80
PF02889	Sec63	0	73	0	0	0	0	0
PF14604	SRC Homology 3 Domain_9	0	39	0	0	0	0	0
PF00083	Sugar transporter	0	3	0	0	0	33	0
PF00957	Synaptobrevin	0	61	0	0	0	0	0
PF01822	cell wall stress response component (WSC)	0	6	0	0	0	17	73
PF00172	Zn(2)-Cys(6) binuclear cluster domain	0	0	0	0	40	0	0
No PFAM	-	100	100	0	100	100	67	100
	number of CRO	*11*	*33*	*1*	*1*	*5*	*6*	*15*

Fig. S1. Distribution of CROs in *Colletotrichum* species and genomic neighborhood. Fungal CROs are today classified in auxiliary activities family 5 (AA5) of the carbohydrate-active enzyme database (*74*). The AA5 family is divided into two evolutionarily divergent sub-families, the AA5_1 containing the glyoxal oxidases (GLOx) and the AA5_2 containing all the other members hitherto reported, including the AlcOx (*19*). **(A)** Phylogenetic analysis of the catalytic domain of all CRO AA5_2 sequences from 30 *Colletotrichum* species. Biochemically characterized AA5_2s are indicated in black and bold face.The *Cor*AlcOx, characterized in this study, is shown in red, and the AA5_2s found in tandem with a putative peroxidase are pinpointed by a green asterisk. **(B)** Distribution of CRO types found for each species (*Cor*AlcOx is framed in red). The full list of *Colletotrichum* species, and

associated abbreviations, are provided in **Table S5**. **(C)** Condensed matrix of AA5_2s neighboring genes. Only PFAM domains occurring with a frequency > 30% within the neighborhood of at least one of the AA5_2 clades are shown. «No PFAM» refers to domains not associated with any PFAM code. The content of the matrix reads as follows: e.g., PFAM 00141 (framed in red) is found in the neighborhood of 100% of the CRO-AlcOx and is not detected for any of the other AA5_2s. A color gradient from white to green representing occurrence frequencies from 0 to 100%, respectively, has been applied. Abbreviation: AlcOx, primary alcohol oxidase; *Cgl*, *Colletotrichum gloeosporioides*; *Cgr*, *Colletotrichum graminicola*; *Fgr*, *Fusarium graminearum*; AAO, aryl alcohol oxidase; RafOx, raffinose oxidase; UNK, unknown.

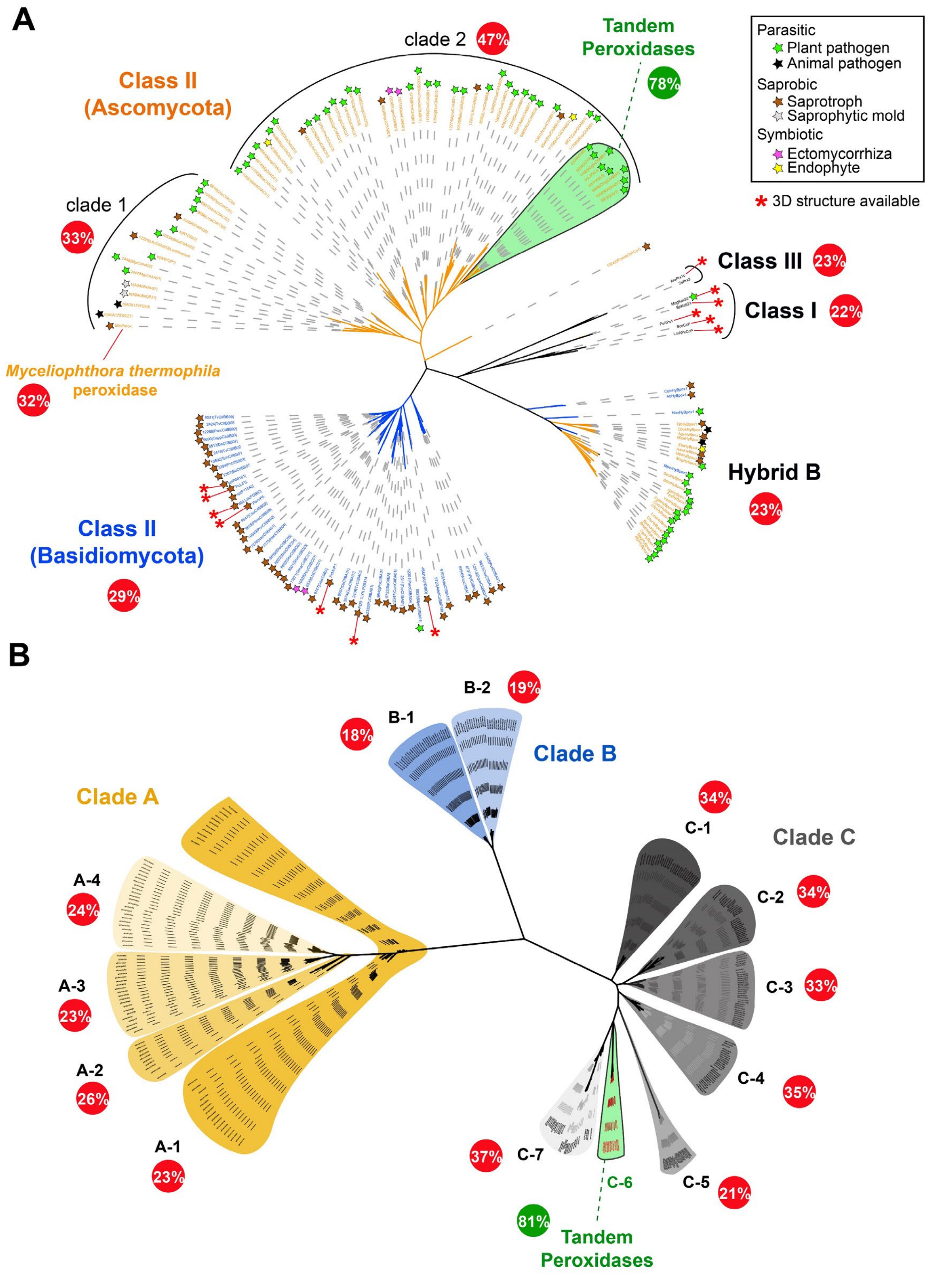

Fig. S2. Phylogenetic analysis of Tandem Peroxidases. (A) Location of the Tandem Peroxidase amongst the peroxidase-catalase superfamily**,** containing peroxidases from Class I (intracellular peroxidases), II (fungal secreted peroxidases) and III (plant secreted peroxidases). The peroxidases and related proteins from Ascomycetes and Basidiomycetes are shown in orange and blue, respectively. The well-known lignin-active peroxidases are found in the Class II-Basidiomycetes clade. Hybrid B peroxidases are fungal peroxidases that form a distinct group from the evolutionary-related Class II peroxidases (*52*). **(B)** Phylogeny analysis of the 333 putative class II peroxidases from 30 *Colletotrichum* species. In both panels, we provide the average sequence identity within the Tandem Peroxidase clade (circled in green) and in between the latter against the other clades (circled in red).

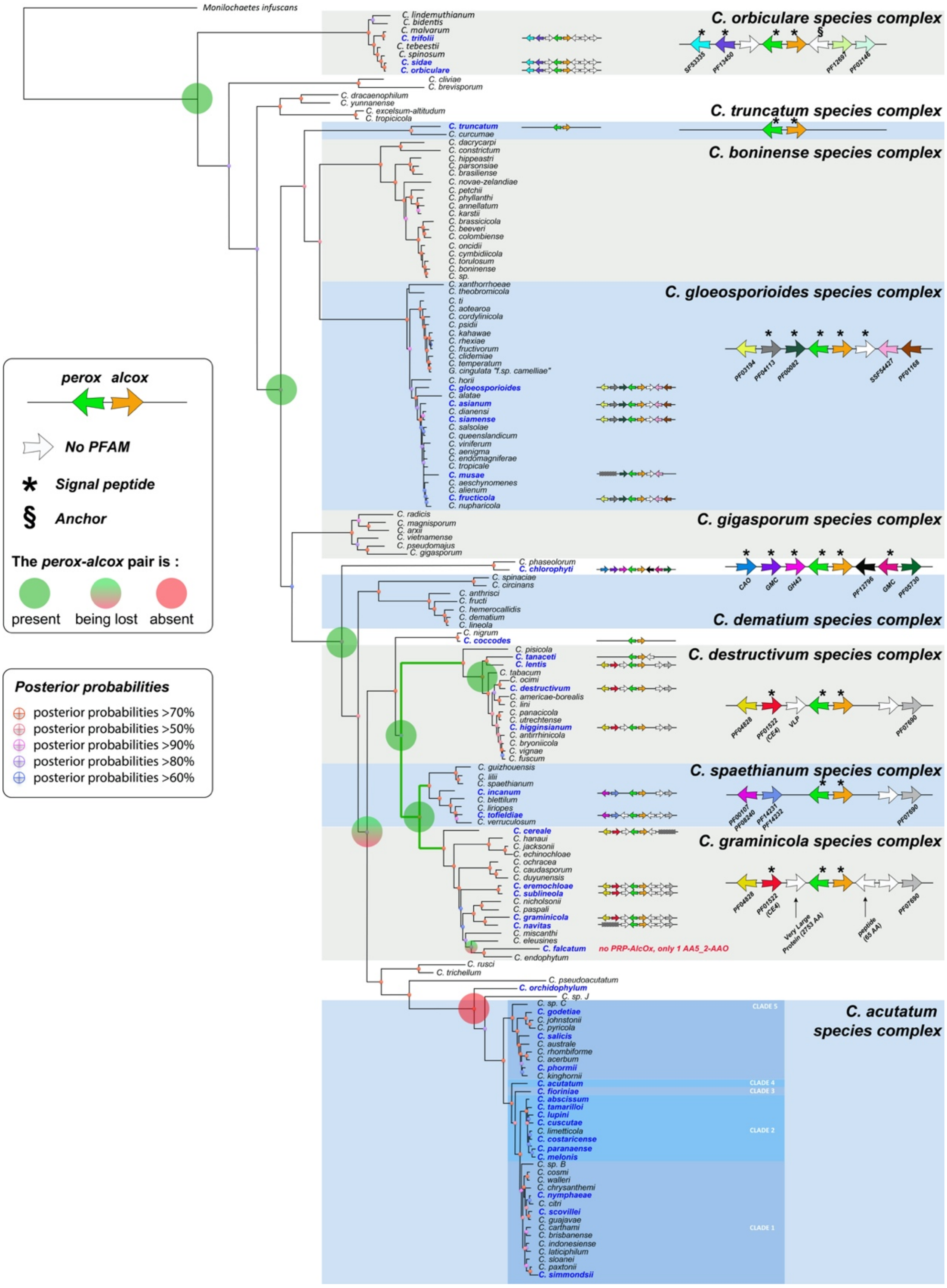

Fig. S3. Mapping of the occurrence of the *perox-alcox* pair and genomic neighborhood on the phylogenomic tree of *Colletotrichum* species. The initial tree was built with 133 *Colletotrichum* species by Baroncelli et al. (*75*). The genomes of species written in dark blue and bold face are available (note the absence of sequenced genomes in the *C. boninense*, *C. gigasporum* and *C. dematium* species complexes). Key to symbols is provided in the figure. For each species complex, consensus gene organization is shown on the right-hand side.

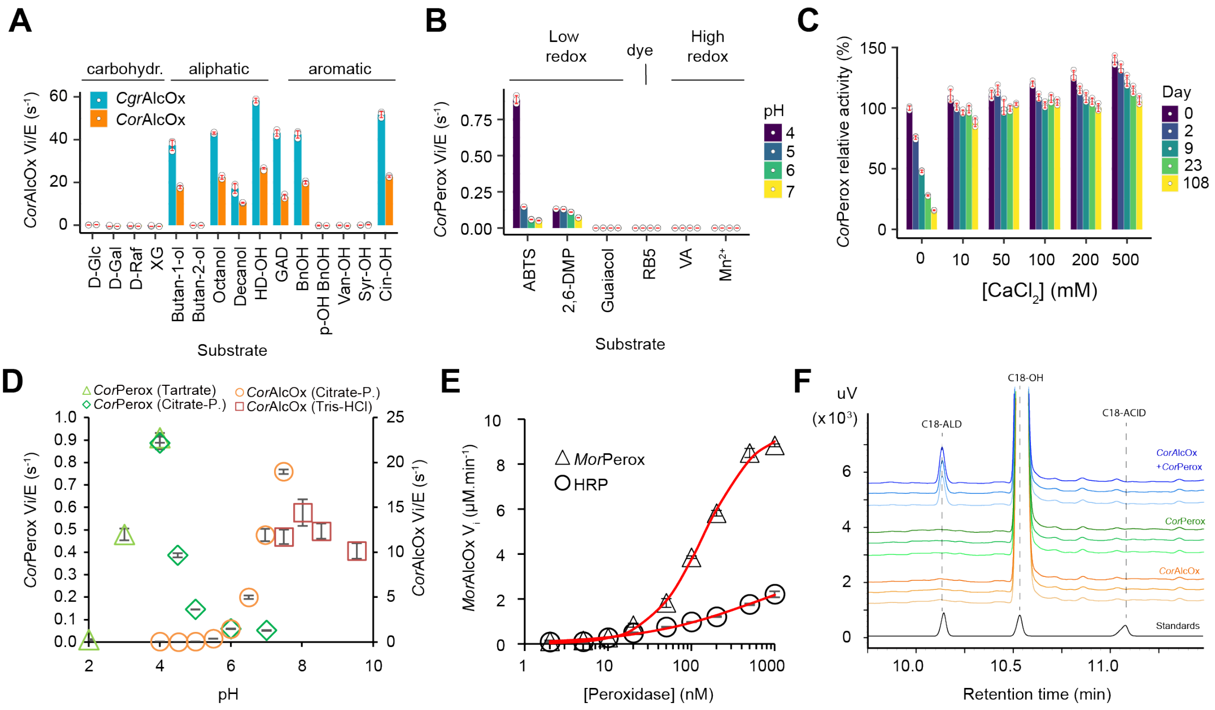

Fig. S4. Biochemical characterization of Tandem AlcOx and Perox. (A) *Cor*AlcOx substrate specificity screening and comparison with the previously characterized AlcOx from *C. graminicola* (*Cgr*AlcOx) (*19*). Substrates abbreviations are provided in the experimental section. **(B)** Activity of *Cor*Perox on different peroxidase substrates. **(C)** Stability of *Cor*Perox in presence of different concentrations of $CaCl_2$ when stored at 4°C. **(D)** pH-dependent activity of *Cor*Perox (on H_2O_2 and ABTS) and *Cor*AlcOx (on BnOH). **(E)** *Mor*AlcOx activation by *Mor*Perox *vs* HRP. **(F)** Gas chromatography analyses of octadecanol (C18-OH) oxidation into *n*-octadecanal (C18-ALD) by *Cor*AlcOx and *Cor*Perox, added individually or in combination. Chromatograms shown with different shades of a same color correspond to independent replicates. In panels **A to E**, data are presented as average values (n = 3 independent biological replicates) and error bars show s.d..

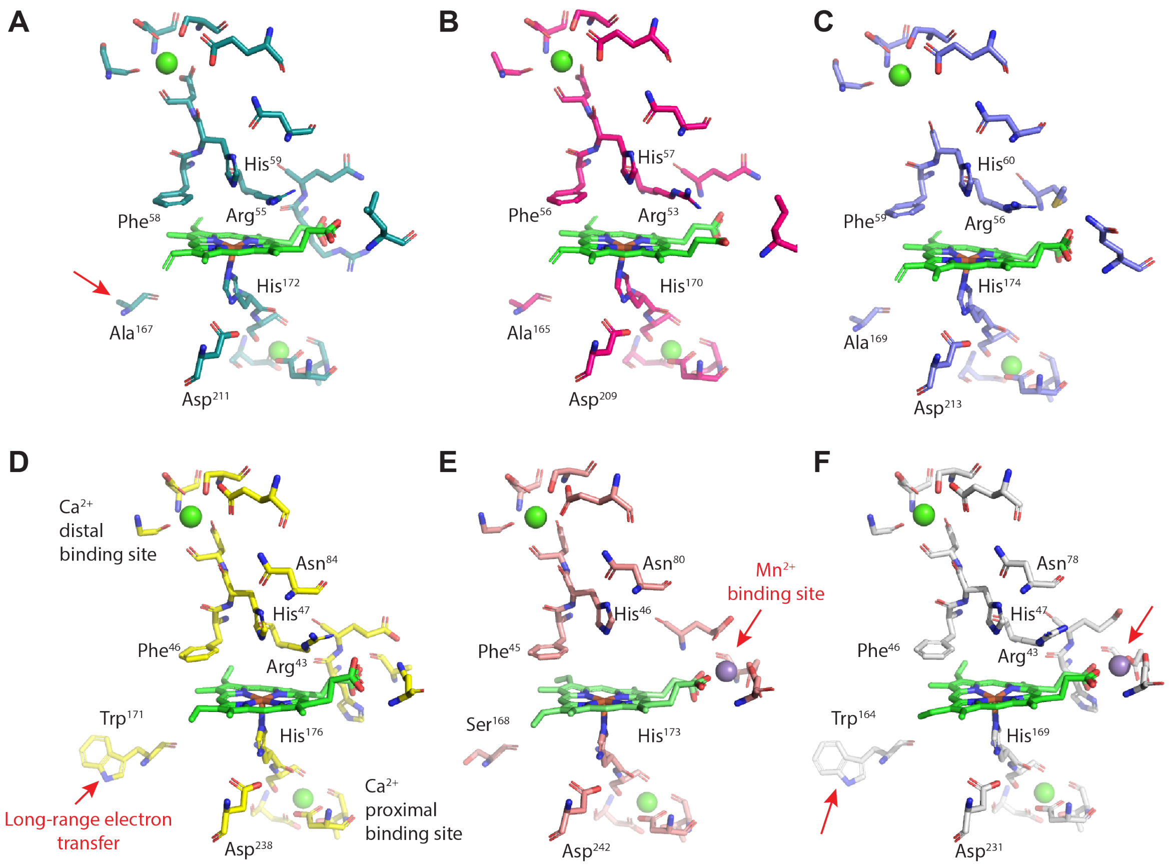

G

	Tandem Peroxidases			Function conserved ?	Lignin-active Peroxidases		
**Functional Role**	(A) *Cgr*Perox	(B) *Cor*Perox	(C) *Mor*Perox		(D) *Pch*LiP	(E) *Pch*MnP	(F) *Per*VP
Long-range e^- transfer	A167	A165	A169	NO	W171	S168	W164
Mn^{2+}-binding Site	S48 Q52 no eq. nd	A46 Q50 no eq. nd	A49 M53 no eq. nd	NO	A36 E40 D182 Prop. (Heme)	E35 E39 D179 Prop. (Heme)	E36 E40 D175 Prop. (Heme)
Ca^{2+} distal binding Site	D60 S65 D67 S69 E75	D58 A63 D65 S67 E73	D61 S66 D68 S70 E76	YES	D48 G66 D68 S70 E78 (via H_2O)	D47 G62 D64 S66 E74 (via H_2O)	D48 G60 D62 S64 E72 (via H_2O)
Ca^{2+} proximal binding Site	S173 D184 T186 D191	S171 D182 S184 D189	S175 D186 T188 D193	YES	S177 D194 T196 D201	S174 D191 T193 D198	S170 D187 T189 D194

Fig. S5. Active site details of homology models of Tandem Peroxidases *vs* crystal structures of well-characterized lignin-active peroxidases. (A to C) Homology models (predicted with alpha-fold algorithm followed by docking of calcium ions and heme group) of Tandem Peroxidases from *C. graminicola* (*Cgr*Perox) **(A)**, *C. orbiculare* (*Cor*Perox) **(B)** and *M. oryzae* (*Mor*Perox) **(C)**. **(D to F)** X-ray crystallographic structures of LiP5 peroxidase from *Phanaerochaete chrysosporium* (PDB 1LLP, *Pch*LiP) (*76*) **(D)**, manganese peroxidase from *P. chrysosporium* (PDB 1MNP, *Pch*MnP) (*77*) **(E)** and versatile peroxidase from *Pleurotus eryngii* (PDB 2BOQ, *Per*VP) (*78*) **(F)**. Only key amino acids involved in catalysis and metal binding are shown. Calcium and manganese atoms are represented by green and purple spheres, respectively. **(G)** Comparison of key residues, categorized according to their functional role, for the Tandem Peroxidases *vs* lignin-active peroxidases. The latter are known to employ two different mechanisms relying on either oxidation by long-range electron transfer via a Trp (LiP) or manganese–catalysed oxidoreduction (MnP), or a combination of both (VP).

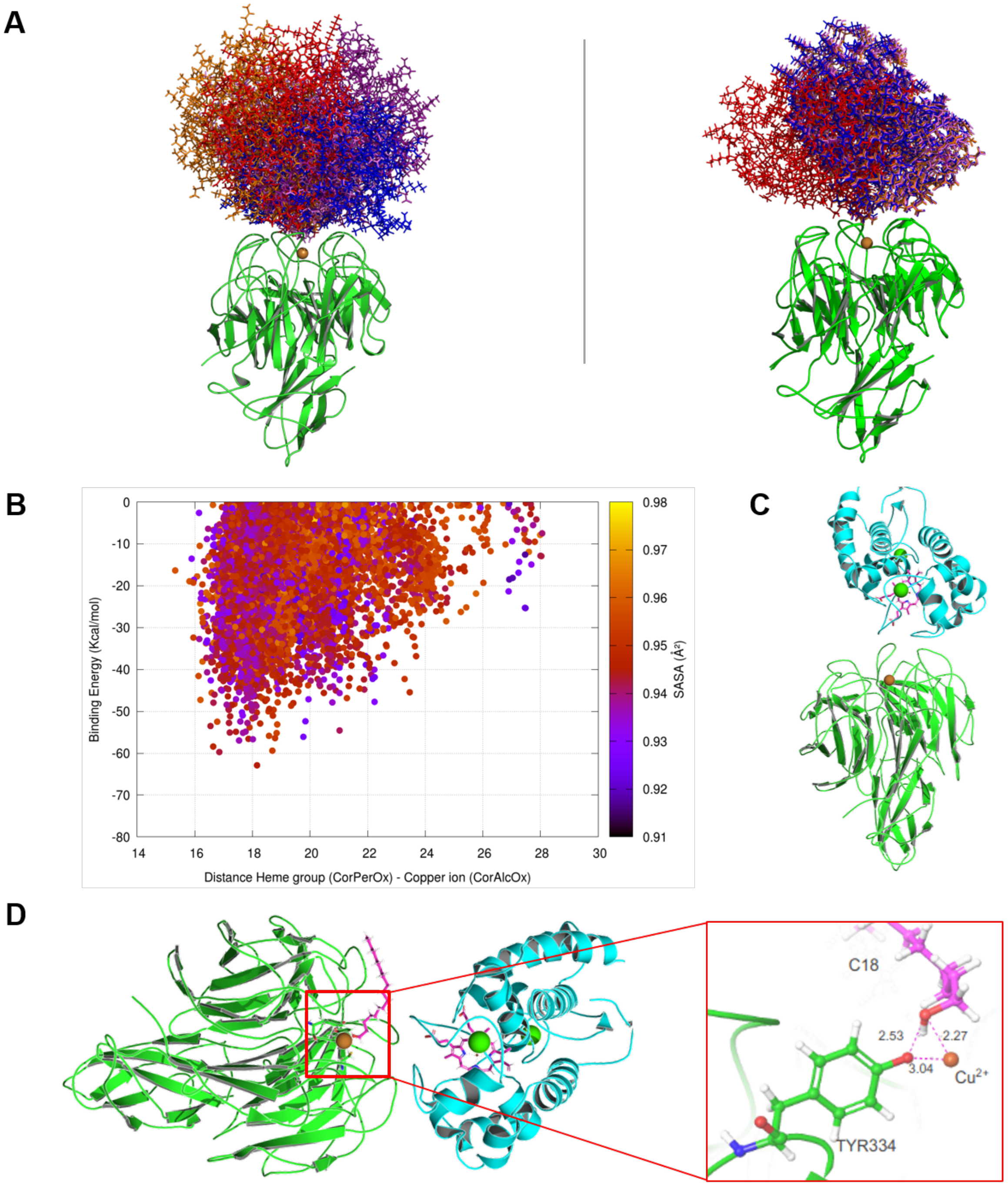

Figure S6. *Cor*AlcOx-*Cor*Perox protein-protein and ligand docking simulation studies. **(A)** Superposition of the representative structures of PIPER's top five clusters (left) and of the five AlphaFold2-Multimer predicted models (right). **(B)** Protein-protein interaction energy as a function of the distance between the heme group (using NA nitrogen atom) of *Cor*Perox and the copper ion of CorAlcOx. The colors indicate the solvent-accessible surface area of *Cor*AlcOx. Each plot point corresponds to a structure obtained during the PELE refinement. **(C)** Minimum energy structure obtained with the PELE refinement (dist(Heme-Cu^{2+})=18.16 Å, Binding Energy=-62.89 kcal/mol). **(D)** Catalytic pose of C18 with *Cor*AlcOx-*Cor*Perox complex found by PELE with a zoom in the catalytic site.

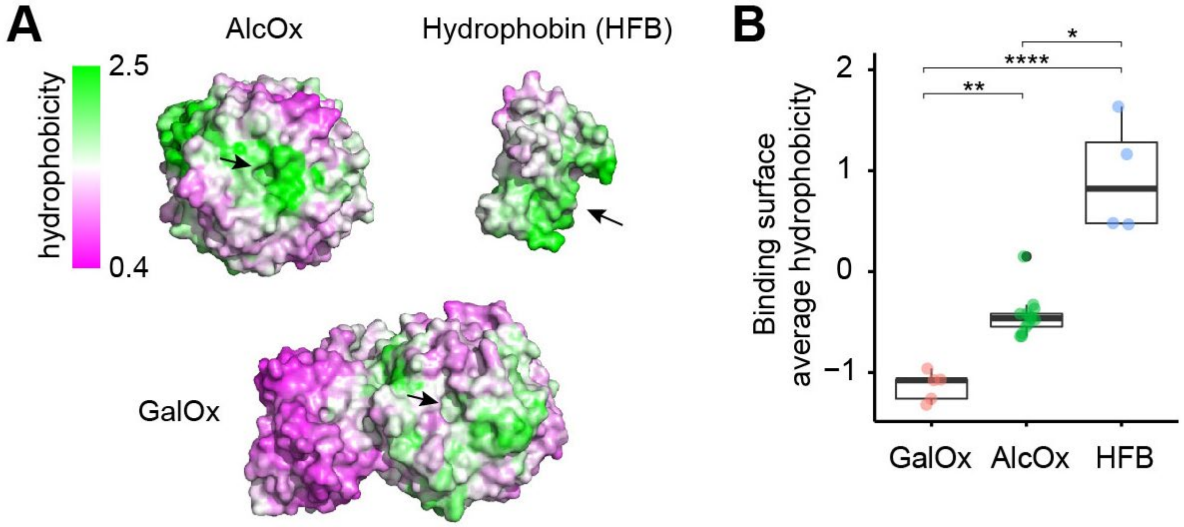

Fig. S7. AlcOx enzymes display a hydrophobic active site. (**A**) Surface hydrophobicity (**B**) and average hydrophobicity (expressed as the grand average of hydropathy (GRAVY) score) of the binding surface (indicated with black arrow) of GalOx (PDB 1GOG) (*79*), AlcOx (PDB 5C92) (*19*) and hydrophobin (HFB, PDB 2N4O) (*80*). In panel B, several orthologs of each protein type were analyzed to yield an average value (n ≥4). *P<0.05, **P<0.01, ****P<0.0001, Kruskal-Wallis test.

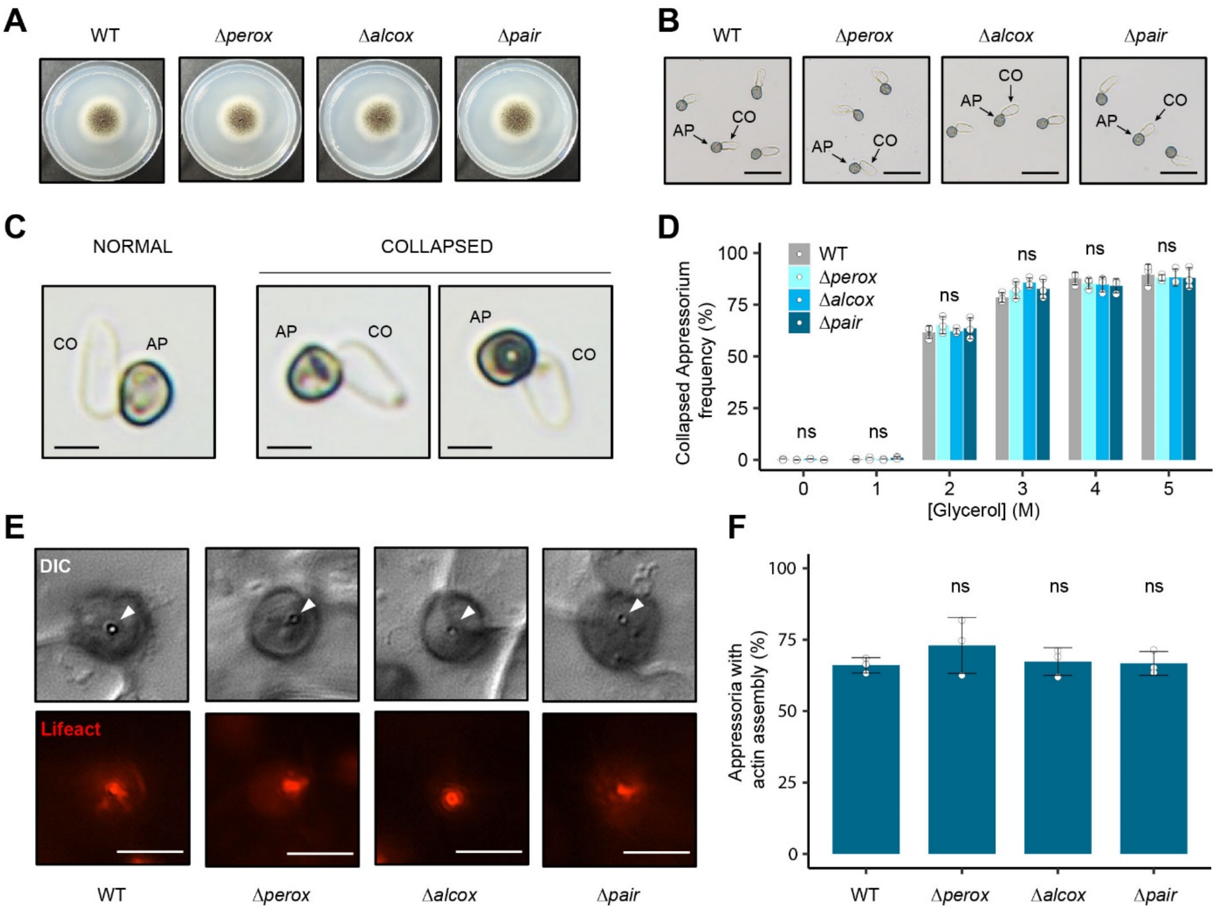

Fig. S8. Unmodified phenotypic features upon deletion of *perox* and/or *alcox* genes. (A and B) *C. orbiculare* WT and *perox/alcox* mutant strains' colonial growth on potato dextrose agar (24°C, 5 days) (**A**) and development of melanized appressorium on glass slides (24 hpi, scale bar = 10 µm) (**B**). (**C and D**) Representative images of normal and collapsed appressorium as indicated by crack and dent in appressorium (scale bar = 5 µm) (**C**) and cytorrhysis assay for appressorial turgor (**D**) for *C. orbiculare* WT and *perox/alcox* mutant strains. Data are presented as average values (>200 appressoria counted for each replicate, n = 3 independent biological replicates) and error bars show s.d.. **(E and F)**, Representative images of actin assembly at the appressorium pore in *C. orbiculare* WT and *perox/alcox* mutant strains carrying Lifeact-RFP (**E**) and corresponding frequency of appressoria that formed an actin assembly at the pore (**F**). In panel **E**, white arrowheads indicate appressorium pore (scale bar = 5 µm). In panel **F**, data are presented as average values (>50 appressoria scored for each replicate, n = 3 independent biological replicates) and error bars show s.d.. In panels **D** and **F**, a two-tailed independent *t*-test *vs* WT was applied for all mutant strains (ns, not significant). Abbreviations: AP, appressorium; CO, conidium; hpi, hour post inoculation.

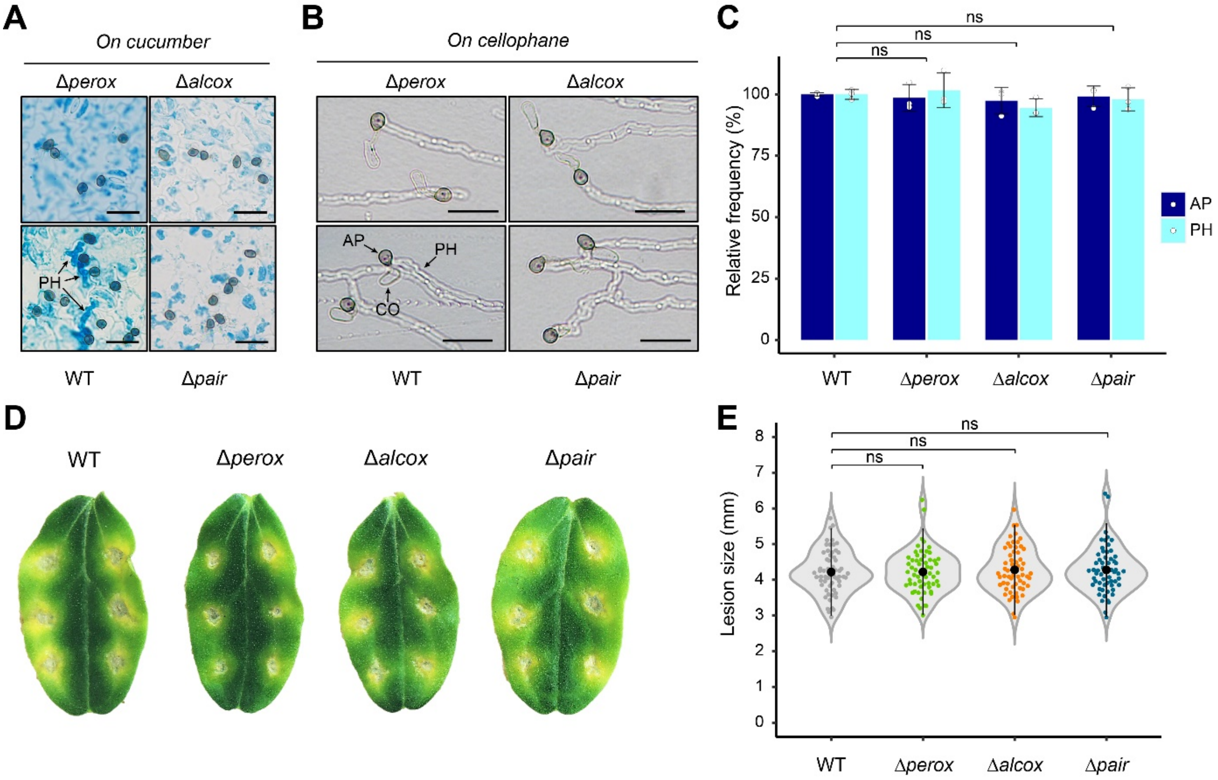

Fig. S9. *Colletotrichum orbiculare* Perox-AlcOx pair is not required for penetrating cellophane and wounded cucumber cotyledons. (A and B), Development of appressorium (AP) and penetration hyphae (PH) on lower surface of detached cucumber cotyledons at 3 days post inoculation (**A**), or on cellophane membrane at 2 days post inoculation (**B**). In panel **A**, penetration hyphae were stained with lactophenol aniline blue. Scale bar = 20 µm. **c**, Relative frequency (WT set to 100%) of development of normal appressorium (AP) and penetrating hyphae (PH) by *C. orbiculare* strains on cellophane. Data are presented as average values (>300 germinated conidia were evaluated for each replicate, n = 3 independent biological replicates) and error bars show s.d. (see **Fig. 3E** in main text for equivalent data acquired on cucumber). Two-tailed independent *t*-test *vs* WT was applied for both AP and PH series (ns, not significant). **(D and E)**, Pathogenic phenotype of *C. orbiculare* strains on wounded cucumber cotyledons (5 dpi, 24°C) (**D**) and violin plot of the distribution of necrotic lesions size (for each strain, n = 60 inoculations) (**E**). Two-tailed independent *t*-test *vs* WT was applied for each strain (ns, not significant).

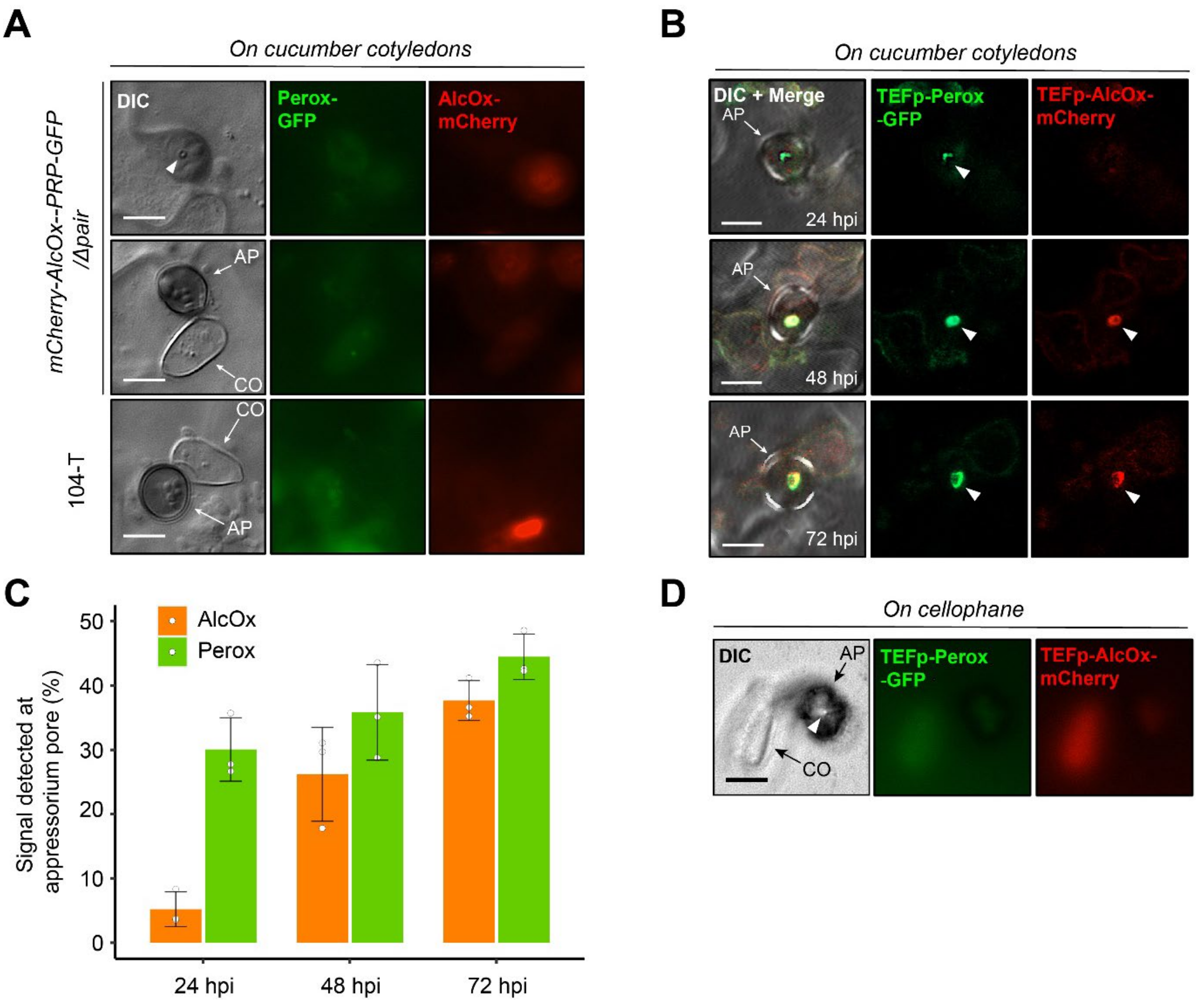

Fig. S10. Fluorescence localization studies of *Cor*AlcOx-mCherry and *Cor*Perox-GFP on either cucumber cotyledons or cellophane, driven by native or *TEF* promoters. (A) Fluorescence microscopy of conidial suspensions of *C. orbiculare* wild-type 104-T strain expressing *CorAlcOx-mCherry* and *CorPerox-GFP* under native promoter. Micrographs were either focused on appressorium pore (top raw) or on appressorium periphery (middle and bottom row). **(B)** Time-course fluorescence microscopy of conidial suspensions of *C. orbiculare* strain expressing *CorAlcOx-mCherry and CorPerox-GFP* under *TEF* promoter. In panels **A** and **B**, conidial suspensions were prepared in distilled water and incubated on lower surface of detached cucumber cotyledons for 24 hpi (**A**) or 24, 48 and 72 hpi (**B**). **(C)** Percentage of detected fluorescence intensity of *Cor*AlcOx-mCherry and *Cor*Perox-GFP localized at appressorium pore. Data are presented as average values (>50 appressoria were analyzed for each replicate, n = 3 independent biological replicates) and error bars show s.d.. **(D)** Fluorescence microscopy of conidial suspensions of *C. orbiculare* expressing *CorAlcOx-mCherry* and *CorPerox-GFP* (under *TEF* promoter) incubated on cellophane membrane for 16 h. In all panels, white arrowheads indicate the appressorium pore; scale bars = 5 µm. Abbreviations: AP, appressorium; CO, conidium; hpi, hour post infection.

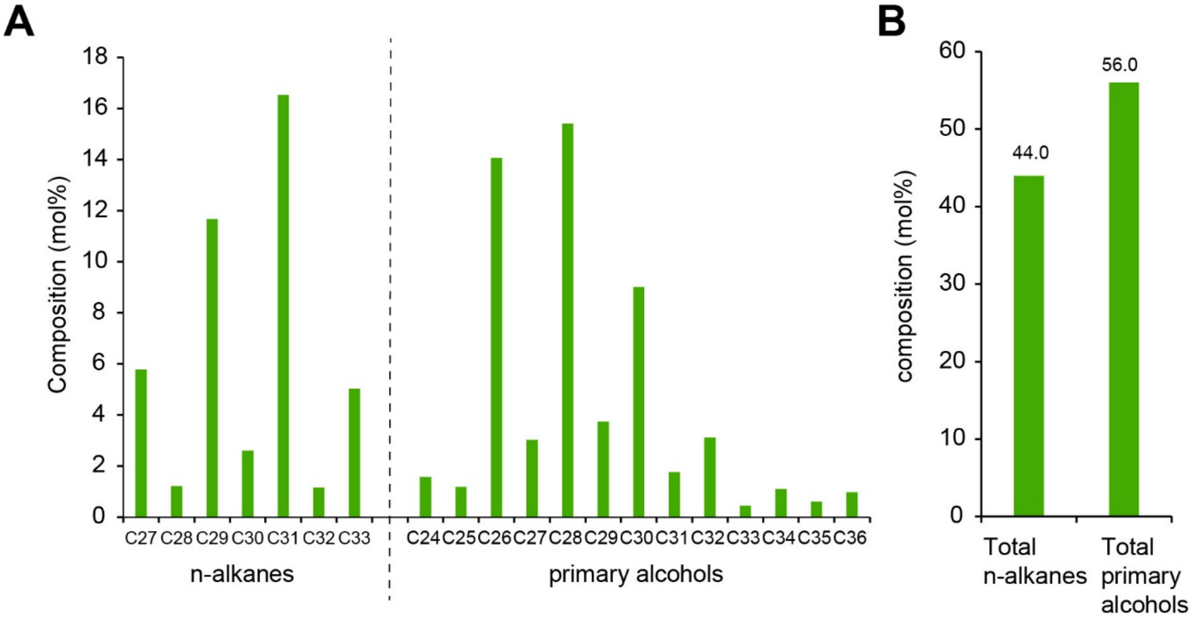

Fig. S11. Compositional analysis of cuticular waxes extracted from cucumber cotyledons. (A) chain-length detail of composition and **(B)** total composition. Extracted waxes were derivatized and analyzed by GC-MS (see material and methods section). Fatty acids, esters, aldehydes and secondary aldehydes were not detected (or as traces)

Table S1. Identification of *perox-alcox* tandems by the "gene neighborhood survey" approach on *Colletotrichum* genomes.

Species name		AlcOx			Head-to-head ?	Tandem Peroxidase		
		**JGI ID**	**Locus**	**SP?**		**JGI ID**	**Locus**	**SP?**
Colletotrichum	Colce1	810512	scaffold_588:1104-2984	YES	YES	607041	scaffold_588:4000-4907	YES
	Colch1	3549	MPGH_1000056:334431-336305	YES	YES	3548	MPGH_1000056:332594-333501	YES
	Coler1	694318	scaffold_85:74252-76309	YES	YES	565608	scaffold_85:76979-78147	YES
	Colgr1	3021	Supercontig_19:380,578-382,421	YES	YES	3020	Supercontig_19:378775-379688	YES
	Colhig2	13170	chromosome_8:3,372,269-3,370,404	YES	YES	13171	chromosome_8:3373256-3374168	YES
	Colin1	4733	KV_841907:44467-46332	YES	YES	4734	KV_841907:47355-48250	YES
	Colna1	705233	scaffold_51:260,365-259,077	NO	YES	671564	scaffold_51:260654-260938	YES
	Colorb1	143883	KB_726046:462,434-460,620	YES	YES	143884	KB_726046:463450-464344	YES
	Colsu1	678444	scaffold_47:124,867-123,016	YES	YES	724645	scaffold_47:125684-126629	YES
	Coltofl	150683	LFHQ_1000160:315,111-313,247	YES	YES	150684	LFHQ_1000160:316125-316900	YES
	Gloci1	1901294	scaffold_25:435,642-437,422	YES	YES	1753690	scaffold_25:433668-434565	YES

Abbreviations : SP, signal peptide

Table S2. Identification of *perox-alcox* tandems by the "cross-genome BLAST" approach[a].

	Species name	AlcOx			Head-to-head ?	Tandem Peroxidase		
		**Gene bank accession ID**	**Seq. Id[b]**	**SP?**		**Gene bank accession ID**	**Seq. Id[c]**	**SP?**
Colletotrichum	*C. orbiculare MAFF 240422*	TDZ17043.1	100 %	YES	YES	TDZ17044.1	100 %	YES
	C. trifolii	TDZ61515.1	99.6 %	YES	YES	TDZ61518.1	98.9 %	YES
	C. chlorophyti	OLN93108.1	85.2 %	YES	YES	OLN93067.1	80.0 %	YES
	C. tanaceti	TKW54882.1	84.6 %	YES	YES	TKW54881.1	79.2 %	YES
	C. fructicola Nara gc5	ELA25906.1	86.4 %	YES	YES	ELA25905.1	78.9 %	YES
	C. higginsianum IMI 349063	XP_018153777.1	84.4 %	YES	YES	XP_018153778.1	78.5 %	YES
	C. sublineola	KDN67444.1	82.8 %	YES	YES	KDN67445.1	76.6 %	YES
	C. tofieldiae	KZL75366.1	84.8 %	YES	YES	KZL75346.1	76.5 %	YES
	C. incanum	KZL83752.1	84.6 %	YES	YES	KZL83755.1	74.2 %	YES
	C. graminicola M1.001	XP_008094466.1	85.3 %	YES	YES	XP_008094465.1	71.8 %	YES
Magnaporthe	*M. oryzae Y34*	ELQ45116.1	65.9 %	YES	YES	ELQ45117.1	63.0 %	YES
	M. pennisetigena	XP_029748983.1	64.3 %	YES	YES	XP_029748984.1	61.2 %	YES
	M. oryzae 70-15	XP_003719369.1	65.9 %	YES	YES	XP_003719370.1	60.8 %	YES
	M. oryzae	QBZ64255.1	65.8 %	YES	YES	QBZ64256.1	60.3 %	YES
	M. sp. CBS 133598	TLD18113.1	65.7 %	YES	YES	TLD18188.1	59.5 %	YES
	M. grisea	XP_030976776.1	66.9 %	YES	YES	XP_030976779.1	58.6 %	YES

[a] Sequence identity thresholds of 60% and 50% were applied for the BLAST searches using *Cor*AlcOx and *Cor*Perox as queries, respectively.
[b] Sequence identity (in %) calculated relatively to *Cor*AlcOx
[c] Sequence identity (in %) calculated relatively to *Cor*Perox
Abbreviations : SP, signal peptide

Table S3. List of *Colletotrichum* species (and abbreviations) used in Fig. S1.

FULL NAME	**Abbreviation**
Colletotrichum acutatum CBS 112980 v2.0	Colac2
Colletotrichum caudatum CBS131602 v1.0	Colca1
Colletotrichum cereale CBS 129662 v1.0	Colce1
Colletotrichum chlorophyti NTL11	Colch1
Colletotrichum costaricense IMI 309622	Colco1
Colletotrichum cuscutae IMI 304802	Colcu1
Colletotrichum eremochloae CBS129661 v1.0	Coler1
Colletotrichum falcatum MAFF306170 v1.0	Colfa1
Colletotrichum fioriniae PJ7	Colfi1
Colletotrichum godetiae CBS 193.32 v1.0	Colgo1
Colletotrichum graminicola M1.001	Colgr1
Colletotrichum higginsianum IMI 349063	Colhig2
Colletotrichum incanum MAFF 238712	Colin1
Colletotrichum lupini CBS 109225 v1.0	Collu1
Colletotrichum melonis CBS 134730	Colme1
Colletotrichum navitas CBS125086 v1.0	Colna1
Colletotrichum nymphaeae SA-01	Colny1
Colletotrichum orchidophilum IMI 309357	Color1
Colletotrichum orbiculare 104-T	Colorb1
Colletotrichum paranaense IMI 384185	Colpa1
Colletotrichum phormii CBS102054 v1.0	Colph1
Colletotrichum salicis CBS607.94	Colsa1
Colletotrichum simmondsii CBS122122	Colsi1
Colletotrichum somersetensis CBS 131599 v1.0	Colso1
Colletotrichum sublineola CBS 131301 v1.0	Colsu1
Colletotrichum tamarilloi CBS 129955	Colta1
Colletotrichum tofieldiae CBS 168.49	Coltof1
Colletotrichum zoysiae MAFF235873 v1.0	Colzo1
Glomerella acutata v1.0	Gloac1
*Glomerella cingulata 23 (Colletotrichum gloeosporoides 23) v1.0**	Gloci1

*today renamed as *Colletotrichum fructicola Nara gc5*

Table S4. Strains of *C. orbiculare* used in this study.

***C. orbiculare* strains**

Strain name	Genotype description	Reference[a]
104-T (MAFF240422)	Wild type	Ref. (*70*)
Δalcox-1	Wild type / *Δalcox*	This study
Δalcox-2	Wild type / *Δalcox*	This study
Δprp-1	Wild type / *Δprp*	This study
Δprp-2	Wild type / *Δprp*	This study
Δpair-1	Wild type / *Δalcox / Δprp*	This study
Δpair-2	Wild type / *Δalcox / Δprp*	This study
Lifeact-RFP / WT	Wild type / *Lifeact-RFP*	Ref. (*80*)
Lifeact-RFP / *Δalcox*	Wild type / *Δalcox / Lifeact-RFP*	This study
Lifeact-RFP / *Δprp*	Wild type / *Δprp / Lifeact-RFP*	This study
Lifeact-RFP / *Δpair*	Wild type / *Δalcox / Δprp / Lifeact-RFP*	This study
AlcOx-mCherry-PRP-GFP / *Δpair*	Wild type / *Δalcox / Δprp / alcox-mCherry-PRP-GFP*	This study
TEF-AlcOx-mCherry / TEF-PRP-GFP / *Δpair*	Wild type / *Δalcox / Δprp / TEF-alcox-mCherry / TEF-PRP-GFP*	This study
Δssd1	Wild type / *Δssd1*	Ref. (*68*)

[a] Reference numbers shown in this Table correspond to reference numbers provided in the main manuscript

Table S5 | List of primers used in this study.

Primer Name	Sequence (5' → 3')	Amplicon	Note
CoA_R_F1	AGTTGATAATGGGAAACCTGGTTATTCTTGGCAGC	CorAlcOx 5' UTR	For CorAlcOx gene deletion vector pPZP-CoA-N
CoA_R_R1	GATTCATTAATGCAGCGGATAGCTTGTCTTGCCTC		
CoA_L_F1	TCGCTATTACGCCAGGTCAACCATCTTCACCGACC	CorAlcOx 3' UTR	
CoA_L_R1	CTGTGGCGTTGGCACGATGTTCGCCATGAACGAGG		
Neo_F1	GTGCCAACGCCACAGTGCCCCACATCTCCCGGCTG	Neomycin-resistance cassette	
Neo_R1	TTCCCATTATCAACTCAGAAGAACTCGTCAAGAAG		
CoP_L_F1	TCGCTATTACGCCAGCAGCATGTTTCCAGACTCCG	CorPerox 5' UTR	For CorPerox gene deletion vector pPZP-CoP-H
CoP_L_R1	AAGCCCAAAAAATGCATCGAGCCAACGAGTAACG C		
CoP_R_F1	ATGCCGACCGGGAACATTGGTTTGATGGACGGAG C	CorPerox 3' UTR	
CoP_R_R1	GATTCATTAATGCAGTTGTTCAAGGACAGGATGCC		
hph_pPZP_F1	GCATTTTTTGGGCTTGGCTG	Hygromycin-resistance cassette	
hph_pPZP_R1	GTTCCCGGTCGGCATCTAC		
CoA_KO_F1	ACCCTTTCTAGGGAATGGGCGTCCACGCAGACAA G	Border sequences of CorAlcOx and left flanking resion	For confirmation of targeted gene deletion of CorAlcOx
CoA_KO_R1	AGAACAAGTTCGACGGCCAGTTCTGGTCGCCGCC G		
CoP_KO_F1	CTCCAAGGTTCCCAACCCAGAGGGGGTGCTTCCCA	Border sequences of CorPRP and right flanking resion	For confirmation of targeted gene deletion of CorPerox
CoP_KO_R1	GCTCAAGCTACGATGCTGGCAACCATTTGGGAAG G		
pPZP_SUR_F1	CTGCATTAATGAATCAACGCCACAGTGCCCCACAT	pPZP-PvuII-SUR Binary vector	For pPZP-CorAlcOx-mCherry-CorPerox-GFP-S plasmid
pPZP_SUR_R1	CTGGCGTAATAGCGAAGAGG		
CoA_mCherry_R1	ACCACCACCACCACCGAGCGCAACCTTGAAGAAC T	pPZP-CoA-CoP-S	
CoA_mCherry_F1-3	CTGTACAGATCTTAAGGTGACATGGAGACGACGA G		
mCherry_F1-3	TTAAGATCTGTACAGCTCGTCCATGCCGCCGGTGG	glymCherry	
40glymCherry_R1	GGTGGTGGTGGTGGTATGGTGAGCAAGGGCGAGG AGGATA		
CoP_GFP_R1	TCCTCCTCCTCCTCCAACCAATTTCGAACAGTCTG	pPZP-CoA-CoP-S	
CoP_GFP_F1	GAGCTGTACAAGTAATGGACGGAGCTGGAGCTGG A		
glyGFPF1	GGAGGAGGAGGAGGAATGGTGAGCAAGGGC	glyGFP	
GFPR1	TTACTTGTACAGCTCGTCCATGCCGAGAGT		
pCAMSUR_F2	TGTGCTGGGGCCGCGCTGGTGGCGTGCGTATTGGC CTGGA	pCAMSUR-TEF Binary vector	For pCAM-TEF-CorPerox-GFP-S plasmid
40TEFpF2	GTTTGACGGTGATGTATGGAAGATGGAGTGAAGT ACGGTT		
CoP-OX_F1	ACATCACCGTCAAACATGCGCTCTTTTAACCAGAT	CorPerox	
CoP-OX_R1	CGCGGCCCCAGCACATTGTTCAAGGACAGGATGC C		
CoA-OX_F1	ACATCACCGTCAAACATGGTCACTCTTTGCTCAAC	CorAlcOx	For pCAM-TEF-CorAlcOx-
CoA-OX_R1	CGCGGCCCCAGCACAGTCAACCATCTTCACCGACC		

			mCherry-S plasmid
CoA_com_F1	TCCCTTAATTCTCCGGTCAACCATCTTCACCGACC	CorAlcOx	For pBI-TEF-CorAlcOx-mCherry-B plasmid
TEFpF1A	CAATCTGATCATGAGGGGTAGCAAACGGTGGTCAA		
pBIG4MRBSrevF1	CTCATGATCAGATTGTCGTTTCCCGCCTTCAGTTT	pBI-G4MRBrev Binary vector	
pBIG4MRBSrevR1	CGGAGAATTAAGGGAGTCACGTTATGACCTCTAGT		

Table S6. AlcOx-Perox pair contributes to regulation of a subset of the plant-inducible genes predicted to encode SSPs, CAZymes and membrane transporters.

Group		GenBank ID	Gene description (from NCBI)	SP[a]	Effector[b]	Fold change: WT *vs.* *Δpair* on cucumber	Fold change: WT cellophane *vs.* cucumber	Phylogeny-guided activity prediction
SSP (small secreted protein; predicted length <300 amino acids)		TDZ16998	guanyl-specific ribonuclease f1	Y	A	-2.00	6.45	Nd
		TDZ17691	hypothetical protein	Y	A	-2.05	4.75	Nd
		TDZ27000	ChEC43	Y	C	-2.07	10.84	Nd
		TDZ17910	hypothetical protein	Y	N	-2.13	5.14	Nd
		TDZ26550	ChEC89	Y	A	-2.18	4.03	Nd
		TDZ20221	ChEC65	Y	A/C dual	-2.22	26.19	Nd
		TDZ26040	hypothetical protein	Y	A	-2.54	164.73	Nd
		TDZ27001	hypothetical protein	Y	A	-2.80	13.63	Nd
		TDZ22418	Isonitrile hydratase	Y	N	-2.85	10.49	Nd
		TDZ22188	hypothetical protein	Y	C	-3.18	17.26	Nd
		TDZ16108	hypothetical protein	Y	A/C dual	-3.55	9.25	Nd
CAZymes	AA7	TDZ19335	berberine-like protein	Y	N	-2.10	21.17	AA7 – Clade IIa (cello-oligosaccharide dehydrogenase)
	GH93	TDZ14280	bnr asp-box repeat domain protein	Y	N	-2.13	7.74	exo-α-L-1,5-arabinanase
	CBM50	TDZ25669	LysM domain-containing protein	Y	A	-2.16	42.93	Chitin-binding
	CBM50	TDZ16280	LysM domain-containing protein	Y	A	-2.28	33.01	Chitin-binding
	PL3	TDZ18862	pectate lyase	Y	N	-2.30	99.19	PL3_2 (Pectate lyase)
	PL1	TDZ22884	pectin lyase	Y	N	-2.68	29.34	PL3_2 (Pectin lyase)
	AA7	TDZ17089	FAD binding domain protein	Y	N	-2.78	30.05	AA7 – Clade I [c] (*nd*)
	CBM50	TDZ16990	membrane-bound lytic murein transglycosylase dprecursor	Y	A	-2.90	25.36	Chitin-binding
	AA9-CBM1	TDZ16856	endoglucanase ii	Y	N	-3.08	15.76	AA9 LPMO Clade 7 [c] (cellulose/ xyloglucan)

**Table 6** *(continued)*.							
Secondary metabolism	TDZ17906	o-methylsterigmatocystin oxidoreductase	Y	N	-2.05	25.10	Nd
MT (membrane transporter)	TDZ22698	MFS maltose	N	N	-2.01	7.88	Nd
	TDZ14211	peptide transporter mtd1	N	N	-2.07	7.39	Nd
	TDZ17086	integral membrane protein	N	N	-2.77	46.75	Nd
	TDZ24863	opt oligopeptide transporter	N	N	-2.98	5.93	Nd

[a] Predicted presence (Y, Yes; N, No) of signal peptide (SP).
[b] Gene products predicted (using EffectorP-fungi 3.0 (*81*)) to be effectors, localized in plant apoplast (A) and/or plant cytoplasm (C); N, non-effector.
[c] according to clades defined by Momeni et al. (*40*) (for AA7s) and by Vaaje-Kolstad et al. (*82*) (for LPMOs).
Nd, substrate specificity not determined.

Movie S1. 3D confocal image stack of *Cor*AlcOx-mCherry and *Cor*Perox-GFP accumulation on cucumber cotyledons. Conidial suspensions of *C. orbiculare* expressing *CorAlcOx -mCherry* and *CorPerox-GFP* driven by *TEF* promoter were incubated on lower surface of detached cucumber cotyledons for 48 hpi. Scale bar = 5 µm. Grid width = 2 µm. Co-localized *Cor*AlcOx-mCherry and *Cor*Perox-GFP appear as a yellow blob (X: Y: Z = 1.87: 2.05: 1.82 µm) in the center of the image. Green blobs correspond to autofluorescence in plant cells. Plant and fungal cell walls are colored in cyan: the inoculated cucumber sample was treated with Calcofluor White M2R for 1 min before observation.

REFERENCES AND NOTES

1. S. Kamoun, N. J. Talbot, M. Tofazzal Islam, Plant health emergencies demand open science: Tackling a cereal killer on the run. *PLOS Biol.* **17**, 1–6 (2019).

2. M. C. Fisher, N. J. Hawkins, D. Sanglard, S. J. Gurr, Worldwide emergence of resistance to antifungal drugs challenges human health and food security. *Science* **360**, 739–742 (2018).

3. R. Dean, J. A. L. Van Kan, Z. A. Pretorius, K. E. Hammond-Kosack, A. Di Pietro, P. D. Spanu, J. J. Rudd, M. Dickman, R. Kahmann, J. Ellis, G. D. Foster, The top 10 fungal pathogens in molecular plant pathology. *Mol. Plant Pathol.* **13**, 414–430 (2012).

4. C. Beimforde, K. Feldberg, S. Nylinder, J. Rikkinen, H. Tuovila, H. Dörfelt, M. Gube, D. J. Jackson, J. Reitner, L. J. Seyfullah, A. R. Schmidt, Estimating the phanerozoic history of the ascomycota lineages: Combining fossil and molecular data. *Mol. Phylogenet. Evol.* **78**, 386–398 (2014).

5. X. Liang, B. Wang, Q. Dong, L. Li, J. A. Rollins, R. Zhang, G. Sun, Pathogenic adaptations of *Colletotrichum* fungi revealed by genome wide gene family evolutionary analyses. *PLOS ONE* **13**, e0196303 (2018).

6. L. S. Ryder, N. J. Talbot, Regulation of appressorium development in pathogenic fungi. *Curr. Opin. Plant Biol.* **26**, 8–13 (2015).

7. J. C. De Jong, B. J. McCormack, N. Smirnoff, N. J. Talbot, Glycerol generates turgor in rice blast. *Nature* **389**, 244–245 (1997).

8. Y. Kubo, I. Furusawa, in *The Fungal Spore and Disease Initiation in Plants and Animals*, G. T. Cole, H. C. Hoch, Eds. (Springer US, 1991), pp. 205–218.

9. Z. Chen, M. C. Silva, C. J. Rodriguesjr, Appressorium turgor pressure of *Colletotrichum kahawae* might have a role in coffee cuticle penetration. *Mycologia* **96**, 1199–1208 (2004).

10. L. S. Ryder, Y. F. Dagdas, M. J. Kershaw, C. Venkataraman, A. Madzvamuse, X. Yan, N. Cruz-Mireles, D. M. Soanes, M. Oses-Ruiz, V. Styles, J. Sklenar, F. L. H. Menke, N. J.

Talbot, A sensor kinase controls turgor-driven plant infection by the rice blast fungus. *Nature* **574**, 423–427 (2019).

11. M. He, J. Su, Y. Xu, J. Chen, M. Chern, M. Lei, T. Qi, Z. Wang, L. S. Ryder, B. Tang, M. Osés-Ruiz, K. Zhu, Y. Cao, X. Yan, I. Eisermann, Y. Luo, W. Li, J. Wang, J. Yin, S. M. Lam, G. Peng, X. Sun, X. Zhu, B. Ma, J. Wang, J. Liu, H. Qing, L. Song, L. Wang, Q. Hou, P. Qin, Y. Li, J. Fan, D. Li, Y. Wang, X. Wang, L. Jiang, G. Shui, Y. Xia, G. Gong, F. Huang, W. Wang, X. Wu, P. Li, L. Zhu, S. Li, N. J. Talbot, X. Chen, Discovery of broad-spectrum fungicides that block septin-dependent infection processes of pathogenic fungi. *Nat. Microbiol.* **5**, 1565–1575 (2020).

12. Y. F. Dagdas, K. Yoshino, G. Dagdas, L. S. Ryder, E. Bielska, G. Steinberg, N. J. Talbot, Septin-mediated plant cell invasion by the rice blast fungus, Magnaporthe oryzae. *Science* **336**, 1590–1595 (2012).

13. S. Kodama, J. Ishizuka, I. Miyashita, T. Ishii, T. Nishiuchi, H. Miyoshi, Y. Kubo, The morphogenesis-related NDR kinase pathway of *Colletotrichum orbiculare* is required for translating plant surface signals into infection-related morphogenesis and pathogenesis. *PLOS Pathog.* **13**, e1006189 (2017).

14. R. O. Rocha, C. Elowsky, N. T. T. Pham, R. A. Wilson, Spermine-mediated tight sealing of the *Magnaporthe oryzae* appressorial pore–Rice leaf surface interface. *Nat. Microbiol.* **5**, 1472–1480 (2020).

15. M. C. Giraldo, B. Valent, Filamentous plant pathogen effectors in action. *Nat. Rev. Microbiol.* **11**, 800–814 (2013).

16. A. Djamei, K. Schipper, F. Rabe, A. Ghosh, V. Vincon, J. Kahnt, S. Osorio, T. Tohge, A. R. Fernie, I. Feussner, K. Feussner, P. Meinicke, Y.-D. Stierhof, H. Schwarz, B. Macek, M. Mann, R. Kahmann, Metabolic priming by a secreted fungal effector. *Nature* **478**, 395–398 (2011).

17. J. A. D. Cooper, W. Smith, M. Bacila, H. Medina, Galactose oxidase from *Polyporus circinatus*, Fr.*. *J. Biol. Chem.* **234**, 445–448 (1959).

18. P. J. Kersten, T. K. Kirk, Involvement of a new enzyme, glyoxal oxidase, in extracellular H_2O_2 production by *Phanerochaete chrysosporium*. *J. Bacteriol.* **169**, 2195–2201 (1987).

19. D. T. Yin, S. Urresti, M. Lafond, E. M. Johnston, F. Derikvand, L. Ciano, J.-G. Berrin, B. Henrissat, P. H. Walton, G. J. Davies, H. Brumer, Structure–Function characterization reveals new catalytic diversity in the galactose oxidase and glyoxal oxidase family. *Nat. Commun.* **6**, 10197 (2015).

20. Y. Mathieu, W. A. Offen, S. M. Forget, L. Ciano, A. H. Viborg, E. Blagova, B. Henrissat, P. H. Walton, G. J. Davies, H. Brumer, Discovery of a fungal copper radical oxidase with high catalytic efficiency toward 5-hydroxymethylfurfural and benzyl alcohols for bioprocessing. *ACS Catal.* **10**, 3042–3058 (2020).

21. S. B. Lee, M. C. Suh, Advances in the understanding of cuticular waxes in *Arabidopsis thaliana* and crop species. *Plant Cell Rep.* **34**, 557–572 (2015).

22. D. Ribeaucourt, B. Bissaro, M. Yemloul, V. Guallar, H. Brumer, F. Lambert, J.-G. Berrin, M. Lafond, Comprehensive insights into the production of long chain aliphatic aldehydes using a copper-radical alcohol oxidase as biocatalyst. *ACS Sustain. Chem. Eng.* **9**, 4411–4421 (2021).

23. K. Parikka, M. Tenkanen, Oxidation of methyl α-D-galactopyranoside by galactose oxidase: Products formed and optimization of reaction conditions for production of aldehyde. *Carbohydr. Res.* **344**, 14–20 (2009).

24. S. M. Forget, F. R. Xia, J. E. Hein, H. Brumer, Determination of biocatalytic parameters of a copper radical oxidase using real-Time reaction progress monitoring. *Org. Biomol. Chem.* **18**, 2076–2084 (2020).

25. A. Zerva, P. Christakopoulos, E. Topakas, Characterization and application of a novel class II thermophilic peroxidase from *Myceliophthora thermophila* in biosynthesis of polycatechol. *Enzyme Microb. Technol.* **75–76**, 49–56 (2015).

26. R. J. O'Connell, M. R. Thon, S. Hacquard, S. G. Amyotte, J. Kleemann, M. F. Torres, U. Damm, E. A. Buiate, L. Epstein, N. Alkan, J. Altmüller, L. Alvarado-Balderrama, C. A. Bauser, C. Becker, B. W. Birren, Z. Chen, J. Choi, J. A. Crouch, J. P. Duvick, M. A. Farman, P. Gan, D. Heiman, B. Henrissat, R. J. Howard, M. Kabbage, C. Koch, B. Kracher, Y. Kubo, A. D. Law, M. H. Lebrun, Y. H. Lee, I. Miyara, N. Moore, U. Neumann, K. Nordström, D. G. Panaccione, R. Panstruga, M. Place, R. H. Proctor, D. Prusky, G. Rech, R. Reinhardt, J. A. Rollins, S. Rounsley, C. L. Schardl, D. C. Schwartz, N. Shenoy, K. Shirasu, U. R. Sikhakolli, K. Stüber, S. A. Sukno, J. A. Sweigard, Y. Takano, H. Takahara, F. Trail, H. C. Van Der Does, L. M. Voll, I. Will, S. Young, Q. Zeng, J. Zhang, S. Zhou, M. B. Dickman, P. Schulze-Lefert, E. V. L. Van Themaat, L. J. Ma, L. J. Vaillancourt, Lifestyle transitions in plant pathogenic *Colletotrichum* fungi deciphered by genome and transcriptome analyses. *Nat. Genet.* **44**, 1060–1065 (2012).

27. P. Gan, K. Ikeda, H. Irieda, M. Narusaka, R. J. O'Connell, Y. Narusaka, Y. Takano, Y. Kubo, K. Shirasu, Comparative genomic and transcriptomic analyses reveal the hemibiotrophic stage shift of *Colletotrichum* fungi. *New Phytol.* **197**, 1236–1249 (2013).

28. Y. Dong, Y. Li, M. Zhao, M. Jing, X. Liu, M. Liu, X. Guo, X. Zhang, Y. Chen, Y. Liu, Y. Liu, W. Ye, H. Zhang, Y. Wang, X. Zheng, P. Wang, Z. Zhang, Global genome and transcriptome analyses of *Magnaporthe oryzae* epidemic isolate 98-06 uncover novel effectors and pathogenicity-related genes, revealing gene gain and lose dynamics in genome evolution. *PLOS Pathog.* **11**, 1–30 (2015).

29. M. Shimizu, Y. Nakano, A. Hirabuchi, K. Yoshino, M. Kobayashi, K. Yamamoto, R. Terauchi, H. Saitoh, RNA-Seq of in planta-expressed *Magnaporthe oryzae* genes identifies MoSVP as a highly expressed gene required for pathogenicity at the initial stage of infection. *Mol. Plant Pathol.* **20**, 1682–1695 (2019).

30. Y. Kubo, Y. Takano, Dynamics of infection-related morphogenesis and pathogenesis in *Colletotrichum orbiculare*. *J. Gen. Plant Pathol.* **79**, 233–242 (2013).

31. J. N. Rodriguez-Lopez, A. T. Smith, R. N. F. Thorneley, Role of arginine 38 in horseradish peroxidase. A critical residue for substrate binding and catalysis. *J. Biol. Chem.* **271**, 4023–4030 (1996).

32. B. Bissaro, A. Varnai, Å. K. Røhr, V. G. H. Eijsink, Oxidoreductases and reactive oxygen species in conversion of lignocellulosic biomass. *Microbiol. Mol. Biol. Rev.* **82**, e00029-18 (2018).

33. I. Ayuso-Fernández, F. J. Ruiz-Dueñas, A. T. Martínez, Evolutionary convergence in lignin-degrading enzymes. *Proc. Natl. Acad. Sci. U.S.A.* **115**, 6428–6433 (2018).

34. S. Oide, Y. Tanaka, A. Watanabe, M. Inui, Carbohydrate-binding property of a cell wall integrity and stress response component (WSC) domain of an alcohol oxidase from the rice blast pathogen *Pyricularia oryzae*. *Enzyme Microb. Technol.* **125**, 13–20 (2019).

35. D. Kozakov, R. Brenke, S. R. Comeau, S. Vajda, PIPER: An FFT-based protein docking program with pairwise potentials. *Proteins Struct. Funct. Bioinform.* **65**, 392–406 (2006).

36. R. Evans, M. O'Neill, A. Pritzel, N. Antropova, A. Senior, T. Green, A. Žídek, R. Bates, S. Blackwell, J. Yim, O. Ronneberger, S. Bodenstein, M. Zielinski, A. Bridgland, A. Potapenko, A. Cowie, K. Tunyasuvunakool, R. Jain, E. Clancy, P. Kohli, J. Jumper, D. Hassabis, Protein complex prediction with AlphaFold-multimer. bioRxiv 2021.10.04.463034 [**Preprint**]. 10 March 2022. https://doi.org/10.1101/2021.10.04.463034.

37. K. W. Borrelli, A. Vitalis, R. Alcantara, V. Guallar, PELE: Protein energy landscape exploration. A novel Monte Carlo based technique. *J. Chem. Theory Comput.* **1**, 1304–1311 (2005).

38. F. Fukada, Y. Kubo, *Colletotrichum* orbiculare regulates cell cycle G1/S progression via a two-component GAP and a GTPase to establish plant infection. *Plant Cell* **27**, 2530–2544 (2015).

39. K. K. Pennerman, G. Yin, J. W. Bennett, Eight-carbon volatiles: Prominent fungal and plant interaction compounds. *J. Exp. Bot.* **73**, 487–497 (2022).

40. M. Haddad Momeni, F. Fredslund, B. Bissaro, O. Raji, T. V. Vuong, S. Meier, T. S. Nielsen, V. Lombard, B. Guigliarelli, F. Biaso, M. Haon, S. Grisel, B. Henrissat, D. H. Welner, E. R. Master, J. G. Berrin, M. Abou Hachem, Discovery of fungal oligosaccharide-oxidising flavo-enzymes with previously unknown substrates, redox-activity profiles and interplay with LPMOs. *Nat. Commun.* **12**, 2132 (2021).

41. J. M. Sanz-Martín, J. R. Pacheco-Arjona, V. Bello-Rico, W. A. Vargas, M. Monod, J. M. Díaz-Mínguez, M. R. Thon, S. A. Sukno, A highly conserved metalloprotease effector enhances virulence in the maize anthracnose fungus *Colletotrichum graminicola*. *Mol. Plant Pathol.* **17**, 1048–1062 (2016).

42. B. Leuthner, C. Aichinger, E. Oehmen, E. Koopmann, O. Müller, P. Müller, R. Kahmann, M. Bölker, P. H. Schreier, A H2O2-producing glyoxal oxidase is required for filamentous growth and pathogenicity in *Ustilago maydis*. *Mol. Genet. Genomics* **272**, 639–650 (2005).

43. A. K. Chaplin, M. L. C. Petrus, G. Mangiameli, M. A. Hough, D. A. Svistunenko, P. Nicholls, D. Claessen, E. Vijgenboom, J. A. R. Worrall, GlxA is a new structural member of the radical copper oxidase family and is required for glycan deposition at hyphal tips and morphogenesis of *Streptomyces lividans*. *Biochem. J.* **469**, 433–444 (2015).

44. T. M. Vandhana, J.-L. Reyre, S. Dangudubiyyam, J.-G. Berrin, B. Bissaro, J. Madhuprakash, On the expansion of biological functions of lytic polysaccharide monooxygenases. *New Phytol.* **233**, 2380–2396 (2022).

45. F. Sabbadin, S. Urresti, B. Henrissat, A. O. Avrova, L. R. J. Welsh, P. J. Lindley, M. Csukai, J. N. Squires, P. H. Walton, G. J. Davies, N. C. Bruce, S. C. Whisson, S. J. McQueen-Mason, Secreted pectin monooxygenases drive plant infection by pathogenic oomycetes. *Science* **373**, 774–779 (2021).

46. M. Pickl, M. Fuchs, S. M. Glueck, K. Faber, The substrate tolerance of alcohol oxidases. *Appl. Microbiol. Biotechnol.* **99**, 6617–6642 (2015).

47. E. Morin, S. Miyauchi, H. San Clemente, E. C. H. Chen, A. Pelin, I. de la Providencia, S. Ndikumana, D. Beaudet, M. Hainaut, E. Drula, A. Kuo, N. Tang, S. Roy, J. Viala, B. Henrissat, I. V. Grigoriev, N. Corradi, C. Roux, F. M. Martin, Comparative genomics of *Rhizophagus irregularis*, *R. cerebriforme*, *R. diaphanus* and *Gigaspora rosea* highlights specific genetic features in Glomeromycotina. *New Phytol.* **222**, 1584–1598 (2019).

48. A. R. Wattam, D. Abraham, O. Dalay, T. L. Disz, T. Driscoll, J. L. Gabbard, J. J. Gillespie, R. Gough, D. Hix, R. Kenyon, D. Machi, C. Mao, E. K. Nordberg, R. Olson, R. Overbeek, G. D. Pusch, M. Shukla, J. Schulman, R. L. Stevens, D. E. Sullivan, V. Vonstein, A. Warren, R. Will, M. J. C. Wilson, H. S. Yoo, C. Zhang, Y. Zhang, B. W. Sobral, PATRIC, the bacterial bioinformatics database and analysis resource. *Nucleic Acids Res.* **42**, D581–D591 (2014).

49. K. Katoh, D. M. Standley, MAFFT multiple sequence alignment software version 7: Improvements in performance and usability. *Mol. Biol. Evol.* **30**, 772–780 (2013).

50. J. Castresana, Selection of conserved blocks from multiple alignments for their use in phylogenetic analysis. *Mol. Biol. Evol.* **17**, 540–552 (2000).

51. A. Stamatakis, RAxML version 8: A tool for phylogenetic analysis and post-analysis of large phylogenies. *Bioinformatics* **30**, 1312–1313 (2014).

52. M. Zámocký, Š. Janeček, C. Obinger, Fungal hybrid B heme peroxidases – Unique fusions of a heme peroxidase domain with a carbohydrate-binding domain. *Sci. Rep.* **7**, 9393 (2017).

53. K. Katoh, J. Rozewicki, K. D. Yamada, MAFFT online service: Multiple sequence alignment, interactive sequence choice and visualization. *Brief. Bioinform.* **20**, 1160–1166 (2018).

54. I. Letunic, P. Bork, Interactive Tree of Life (iTOL) v4: Recent updates and new developments. *Nucleic Acids Res.* **47**, W256–W259 (2019).

55. J. Jumper, R. Evans, A. Pritzel, T. Green, M. Figurnov, O. Ronneberger, K. Tunyasuvunakool, R. Bates, A. Žídek, A. Potapenko, A. Bridgland, C. Meyer, S. A. A. Kohl, A. J. Ballard, A. Cowie, B. Romera-Paredes, S. Nikolov, R. Jain, J. Adler, T. Back, S. Petersen, D. Reiman, E. Clancy, M. Zielinski, M. Steinegger, M. Pacholska, T. Berghammer, S. Bodenstein, D. Silver, O. Vinyals, A. W. Senior, K. Kavukcuoglu, P. Kohli, D. Hassabis, Highly accurate protein structure prediction with AlphaFold. *Nature* **596**, 583–589 (2021).

56. M. Hebditch, J. Warwicker, Web-based display of protein surface and pH-dependent properties for assessing the developability of biotherapeutics. *Sci. Rep.* **9**, 1–9 (2019).

57. J. Kyte, R. F.Doolittle, A simple method for displaying the hydropathic character of a protein. *J. Mol. Biol.* **157**, 105–132 (1982).

58. G. Madhavi Sastry, M. Adzhigirey, T. Day, R. Annabhimoju, W. Sherman, Protein and ligand preparation: Parameters, protocols, and influence on virtual screening enrichments. *J. Comput. Aided Mol. Des.* **27**, 221–234 (2013).

59. M. H. M. Olsson, C. R. SØndergaard, M. Rostkowski, J. H. Jensen, PROPKA3: Consistent treatment of internal and surface residues in empirical pK_a Predictions. *J. Chem. Theory Comput.* **7**, 525–537 (2011).

60. J. F. Gilabert, D. Lecina, J. Estrada, V. Guallar, Monte Carlo techniques for drug design: The success case of PELE, in *Methods and Principles in Medicinal Chemistry* (John Wiley & Sons Ltd., 2018), pp. 87–103.

61. N. Metropolis, A. W. Rosenbluth, M. N. Rosenbluth, A. H. Teller, E. Teller, Equation of state calculations by fast computing machines. *J. Chem. Phys.* **21**, 1087–1092 (1953).

62. R. A. Friesner, J. L. Banks, R. B. Murphy, T. A. Halgren, J. J. Klicic, D. T. Mainz, M. P. Repasky, E. H. Knoll, M. Shelley, J. K. Perry, D. E. Shaw, P. Francis, P. S. Shenkin, Glide: A new approach for rapid, accurate docking and scoring. 1. Method and assessment of docking accuracy. *J. Med. Chem.* **47**, 1739–1749 (2004).

63. M. Haon, S. Grisel, D. Navarro, A. Gruet, J. G. Berrin, C. Bignon, Recombinant protein production facility for fungal biomass-degrading enzymes using the yeast *Pichia pastoris*. *Front. Microbiol.* **6**, 1–12 (2015).

64. M. M. Bradford, A rapid and sensitive method for the quantitation of microgram quantities of protein utilizing the principle of protein-dye binding. *Anal. Biochem.* **72**, 248–254 (1976).

65. M. J. Martínez, F. J. Ruiz-Dueñas, F. Guillén, Á. T. Martínez, Purification and catalytic properties of two manganese peroxidase isoenzymes from *Pleurotus eryngii*. *Eur. J. Biochem.* **237**, 424–432 (1996).

66. S. Stoll, A. Schweiger, EasySpin, a comprehensive software package for spectral simulation and analysis in EPR. *J. Magn. Reson.* **178**, 42–55 (2006).

67. R. Ménard, G. Verdier, M. Ors, M. Erhardt, F. Beisson, W. H. Shen, Histone H2B monoubiquitination is involved in the regulation of cutin and wax composition in *Arabidopsis thaliana*. *Plant Cell Physiol.* **55**, 455–466 (2014).

68. S. Tanaka, K. Yamada, K. Yabumoto, S. Fujii, A. Huser, G. Tsuji, H. Koga, K. Dohi, M. Mori, T. Shiraishi, R. O'Connell, Y. Kubo, *Saccharomyces cerevisiae* SSD1 orthologues are essential for host infection by the ascomycete plant pathogens *Colletotrichum lagenarium* and *Magnaporthe grisea*. *Mol. Microbiol.* **64**, 1332–1349 (2007).

69. M. Kimura, K. Izawa, K. Yoneyama, T. Arie, T. Kamakura, A novel transformation system for *Pyricularia oryzae*: Adhesion of regenerating fungal protoplasts to collagen-coated dishes. *Biosci. Biotechnol. Biochem.* **59**, 1177–1180 (1995).

70. N. Ishida, S. Akai, Relation of temperature to germination of conidia and appressorium formation in *Colletotrichum lagenarium*. *Mycologia* **61**, 382–386 (1969).

71. K. Saitoh, M. Nishimura, Y. Kubo, N. Hayashi, E. Minami, Y. Nishizawa, Construction of a binary vector for knockout and expression analysis of rice blast fungus genes. *Biosci. Biotechnol. Biochem.* **72**, 1380–1383 (2008).

72. A. J. Vanden Wymelenberg, D. Cullen, R. N. Spear, B. Schoenike, J. H. Andrews, Expression of green fluorescent protein in *Aureobasidium pullulans* and quantification of the fungus on leaf surfaces. *Biotechniques* **23**, 686–690 (1997).

73. G. Tsuji, S. Fujii, S. Tsuge, T. Shiraishi, Y. Kubo, The Colletotrichum lagenarium Ste12-like Gene CST1Is essential for appressorium penetration. *Mol. Plant Microbe Interact.* **16**, 315–325 (2003).

74. A. Levasseur, E. Drula, V. Lombard, P. M. Coutinho, B. Henrissat, Expansion of the enzymatic repertoire of the CAZy database to integrate auxiliary redox enzymes. *Biotechnol. Biofuels* **6**, 41 (2013).

75. R. Baroncelli, D. B. Amby, A. Zapparata, S. Sarrocco, G. Vannacci, G. Le Floch, R. J. Harrison, E. Holub, S. A. Sukno, S. Sreenivasaprasad, M. R. Thon, Gene family expansions and contractions are associated with host range in plant pathogens of the genus *Colletotrichum*. *BMC Genomics* **17**, 1–17 (2016).

76. T. Choinowski, W. Blodig, K. H. Winterhalter, K. Piontek, The crystal structure of lignin peroxidase at 1.70 Å resolution reveals a hydroxy group on the C β of tryptophan 171: A novel radical site formed during the redox cycle 1 1 Edited by R. Huber. *J. Mol. Biol.* **286**, 809–827 (1999).

77. M. Sundaramoorthy, K. Kishi, M. H. Gold, T. L. Poulos, Preliminary crystallographic analysis of manganese peroxidase from *Phanerochaete chrysosporium*. *J. Mol. Biol.* **238**, 845–848 (1994).

78. M. Pérez-Boada, F. J. Ruiz-Dueñas, R. Pogni, R. Basosi, T. Choinowski, M. J. Martínez, K. Piontek, A. T. Martínez, Versatile peroxidase oxidation of high redox potential aromatic compounds: Site-directed mutagenesis, spectroscopic and crystallographic investigation of three long-range electron transfer pathways. *J. Mol. Biol.* **354**, 385–402 (2005).

79. N. Ito, S. E. V Phillips, C. Stevens, Z. B. Ogel, M. J. McPherson, J. N. Keen, K. D. S. Yadav, P. F. Knowles, Novel thioether bond revealed by a 1.7 Å crystal structure of galactose oxidase. *Nature* **350**, 87–90 (1991).

80. C. L. L. Pham, A. Rey, V. Lo, M. Soulès, Q. Ren, G. Meisl, T. P. J. Knowles, A. H. Kwan, M. Sunde, Self-assembly of MPG1, a hydrophobin protein from the rice blast fungus that forms functional amyloid coatings, occurs by a surface-driven mechanism. *Sci. Rep.* **6**, 25288 (2016).

81. J. Sperschneider, P. N. Dodds, EffectorP 3.0: Prediction of Apoplastic and cytoplasmic effectors in fungi and oomycetes. *Mol. Plant Microbe Interact.* **35**, 146–156 (2022).

82. G. Vaaje-Kolstad, Z. Forsberg, J. S. Loose, B. Bissaro, V. G. Eijsink, Structural diversity of lytic polysaccharide monooxygenases. *Curr. Opin. Struct. Biol.* **44**, 67–76 (2017).
